# Sequential Diels–Alder/[3,3]-sigmatropic rearrangement reactions of β-nitrostyrene with 3-methyl-1,3-pentadiene

**DOI:** 10.3762/bjoc.9.251

**Published:** 2013-10-17

**Authors:** Peter A Wade, Alma Pipic, Matthias Zeller, Panagiota Tsetsakos

**Affiliations:** 1 Department of Chemistry, Drexel University, Philadelphia, PA 19104, U.S.A; 2 Department of Chemistry, Youngstown State University, Youngstown, OH, U.S.A

**Keywords:** cycloaddition, diene, nitro, nitronate, rearrangement

## Abstract

The tin(IV)-catalyzed reaction of β-nitrostyrene with (*E*)*-*3-methyl-1,3-pentadiene in toluene afforded two major nitronic ester cycloadducts in 27% and 29% yield that arise from the reaction at the less substituted diene double bond. Also present were four cycloadducts from the reaction at the higher substituted diene double bond, two of which were the formal cycloadducts of (*Z*)*-*3-methyl-1,3-pentadiene. A Friedel–Crafts alkylation product from the reaction of the diene, β-nitrostyrene, and toluene was also obtained in 10% yield. The tin(IV)-catalyzed reaction of β-nitrostyrene with (*Z*)*-*3-methyl-1,3-pentadiene in dichloromethane afforded four nitronic ester cycloadducts all derived from the reaction at the higher substituted double bond. One cycloadduct was isolated in 45% yield and two others are formal adducts of the *E*-isomer of the diene. The product formation in these reactions is consistent with a stepwise mechanism involving a zwitterionic intermediate. The initially isolated nitronic ester cycloadducts underwent tin(IV)-catalyzed interconversion, presumably via zwitterion intermediates. Cycloadducts derived from the reaction at the less substituted double bond of (*E*)*-*3-methyl-1,3-pentadiene underwent a [3,3]-sigmatropic rearrangement on heating to afford 4-nitrocyclohexenes. Cycloadducts derived from the reaction at the higher substituted diene double bond of either diene failed to undergo a thermal rearrangement. Rates and success of the rearrangement are consistent with a concerted mechanism possessing a dipolar transition state. An initial assessment of substituent effects on the rearrangement process is presented.

## Introduction

We have previously reported examples of a general new [3,3]-sigmatropic rearrangement, the conversion of *O*-allyl nitronic esters (nitronates) to γ,δ-unsaturated nitro compounds. In the reported examples, 6-membered cyclic *O*-allyl nitronates were converted to 4-nitrocyclohexenes [1–2]. This rearrangement strongly resembles the classic Claisen rearrangement of allyl vinyl ethers [3–6]. The *O*-allyl nitronic ester rearrangement has been the subject of several theoretical investigations [7–10]. The necessary nitronic ester precursors are readily preparable by Lewis acid-promoted [4 + 2]-cycloaddition reactions of nitroalkenes with appropriate dienes, a general reaction first reported by Denmark and coworkers (Denmark Diels–Alder reaction) [1,11–12]. Other methods of nitronic ester preparation are known and ongoing studies in our laboratory directed at an application to *O*-allyl nitronic ester synthesis will be reported elsewhere. In continuing our investigations, we desired additional *O*-allyl nitronic esters with varied substitution patterns. We were also interested in determining the double bond specificity of the Denmark Diels–Alder reaction for unsymmetrical dienes. Previous work on cyclopentadiene and cyclohexadiene has shown that the reaction can be quite specific to give one of two possible regioisomers [11–12]. In the case of 1-(2-propenyl)cyclohexene only one of four possible regioisomers was observed [1]. However, it was not clear what the outcome would be for a simple open-chain conjugated diene. Consequently, cycloaddition reactions of 3-methyl-1,3-pentadiene were examined. This diene has both trisubstituted and monosubstituted double bonds allowing for cycloaddition to give up to four regioisomers. It is commercially available both as an *E*,*Z-*mixture and as the pure *E*-isomer. The *Z*-isomer can be obtained from the *E*,Z-mixture by selective removal of the *E*-isomer via cycloaddition with maleic anhydride [13].

## Results and Discussion

### Cycloaddition studies

The tin(IV) chloride-catalyzed reaction of β-nitrostyrene and an *E*,*Z*–mixture of 3-methyl-1,3-pentadiene (**1a**,**b**, 70:30, respectively) in dichloromethane solution afforded a series of six Diels–Alder nitronic ester cycloadducts, none of which heavily predominated (Table 1). Only cycloadducts resulting from C–C bond formation at the end of the conjugated diene system were observed, in keeping with reported results for other conjugated dienes [1,11–12]. However, cycloaddition was relatively indiscriminant at the two double bonds of the diene in this initial experiment.

**Table 1:** Cycloaddition products for Sn(IV)-catalyzed reaction of 3-methyl-1,3-pentadiene with β-nitrostyrene.

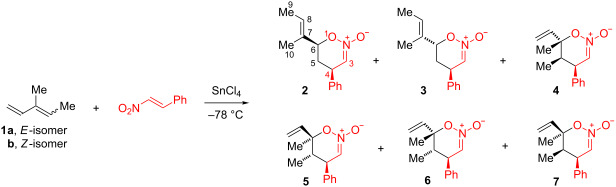							

diene	solvent	ratio^a^ (yield, %)					
		**2**	**3**	**4**	**5**	**6**	**7**

**1a**/**1b**^b^	CH_2_Cl_2_	24 (14)	25 (15)	6 (3)	12 (7)	8 (5)	26 (15)
**1a**	CH_2_Cl_2_	29 (16)	28 (15)	9 (5)	11 (6)	<1	23 (13)
**1b**^c^	CH_2_Cl_2_			2 (1)	6 (4)	19 (12)	73 (45)
**1a**^d^	PhMe	43 (27)	47 (29)	3 (2)	6 (4)	0.5 (0.3)	0.4 (0.2)

^a^Determined by ^1^H NMR. ^b^70:30 ratio. ^c^Adduct **13** (9% yield) also obtained. ^d^Ternary adduct **12a**,**b** (10% yield) also obtained.

The reaction with pure *E*-diene **1a** (*E*/*Z*, 99.7:0.03) was investigated in the hope of eliminating products derived from the *Z*-diene. Here, five cycloadducts were detected. Cycloadducts **2** and **3**, derived from the reaction at the less substituted diene double bond, predominated and were isolated in 16% and 15% yield, respectively. Cycloadduct **7** which is a formal adduct of *Z*-diene **1b** was the third most prevalent product, being formed in 13% yield. Thus, the *E*-diene stereochemistry in this experiment was not retained in the cycloaddition products. It was initially thought that the diene might isomerize under the reaction conditions leading to loss of stereointegrity. However, a more likely explanation is that cycloaddition occurs by a stepwise process involving a tin(IV)-coordinated zwitterion intermediate.

**Scheme 1:**
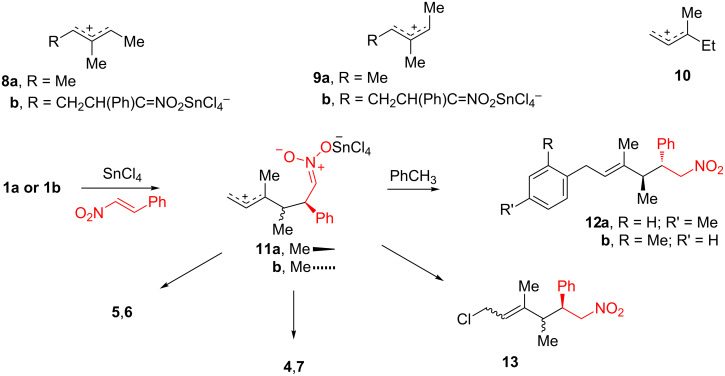
Reaction intermediates, resulting products, and model cations.

The reaction with pure *Z*-diene **1b** gave significantly different results. Only four cycloadducts were obtained, all of which were derived from the attack at the higher substituted diene double bond. Cycloadduct **7** predominated and was isolated in 45% yield. Significantly, the crossover cycloadducts **4** and **5** (formal adducts of the *E*-diene **1a**) were also obtained, albeit in low yield. Here, too, diene stereointegrity was not retained. An open-chain adduct of β-nitrostyrene and the diene was also obtained in 9% yield (vide infra, Scheme 1). The absence of crossover cycloadducts **2** and **3** suggests that diene isomerization is not responsible for the loss of stereointegrity.

Two alternative mechanisms for tin(IV)-catalyzed nitroalkene Diels–Alder reactions have been proposed. Denmark et al. favor a stepwise process proceeding via a zwitterion intermediate [11,14]. Alkene stereointegrity is normally retained, presumably owing to a cyclic conformation dictated by charge interaction between the cation center and tin nitronate, but products derived from carbocation rearrangement of the intermediate have been isolated. Houk et al. favor a concerted mechanism with no intervening energy minimum for cycloaddition based on calculational results [7–9]. The current results are in better agreement with the stepwise mechanism although a competing concerted mechanism is certainly possible. The loss of stereointegrity is not due to simple diene isomerization since cycloadducts **2** and **3** were absent from the product spread derived from *Z*-diene **1b**. The difference in regioselectivity for reactions of *E*-diene **1a** and *Z*-diene **1b** can be rationalized on the basis of the relative stability of allyl cations. The (*E,E*)- and (*E,Z*)-1,2,3-trimethylallyl cations **8a** and **9a** serve as models for projected intermediates **8b** and **9b**, respectively. A calculational study indicates a lower heat of formation for *E,E*-cation **8a** than for *E,Z*-cation **9a** (Δ*H*_f_ is 2.3–3 kcal/mol more favorable for **8a**) [15]. The analogous intermediate **8b** formed from *E*-diene **1a** leads to formation of cycloadducts **2** and **3**. The *Z*-diene **1b** should lead to intermediate **9b** structurally analogous to *E,Z*-cation **9a**. Presumably the higher heat of formation of **9b** is responsible for the absence of products from reaction at the less substituted double bond of diene **1b**. However, it is somewhat surprising that *E*-diene **1a** actually affords cycloadducts **4–7**. We note that model cation **10** (structurally similar to **11a**,**b**) has a higher heat of formation than **8a** (Δ*H*_f_ is 1.8–5.2 kcal/mol more favorable for **8a**) [15]. Nevertheless cycloadducts **4**,**7** and **5**,**6** are formed, presumably via intermediates **11a** and **11b**, respectively.

When the reaction was conducted using pure *E*-diene in toluene solution, the cycloadducts **2** and **3** derived from the reaction at the less substituted double bond were isolated in 27% and 29% yield, respectively. These cycloadducts are sufficiently major to make the cycloaddition a useful process. Cycloadducts **4** and **5** derived from cycloaddition to the higher substituted double bond of *E*-diene **1a** and crossover cycloadducts **6** and **7** were also obtained, all in low yield.

Also present in reactions run in toluene was the ternary adduct **12a**,**b** in which one molecule each of β-nitrostyrene, diene, and toluene was incorporated. This material was isolated in 10% yield as an inseparable 70:30 mixture in which the *p*-isomer predominated. Some partial enrichment of the *p*-isomer was possible through combined chromatographic and recrystallization methods. The structure of nitro compound **12a**,**b** is based on spectral data. However, isomer identification required single-crystal X-ray diffraction analysis, performed on the enriched sample (**12a,b** 91.4:8.6 ratio, respectively). The ternary adduct **12a**,**b** is a Friedel–Crafts alkylation product of toluene and the zwitterion **11a**. Zwitterion **11a** would also lead to cycloadducts **4** and **7** if it cyclized. Possibly zwitterion **11a** is less prone to cyclization than zwitterion **11b** which leads to cycloadducts **5** and **6**. The yield of cycloadduct **7** is very low in experiments where ternary adduct **12a**,**b** is formed. Consistent with the presence of these zwitterions, the related open-chain adduct **13** was a minor product obtained from the reaction of *Z*-diene **1b** and β-nitrostyrene in dichloromethane solution.

### Nitronic ester structure determination

Structure assignments for nitronic esters **2–7** are based on spectral data. The C=N stretching frequencies of several similar 6-membered cyclic nitronic esters were reported to be 1592–1622 cm^−1^ [12,14,16]. Table 2 lists the values for nitronic esters obtained here. Reported ^1^H NMR chemical shifts for HC=N signals (the nitronic H-3 signal) of several cyclic nitronic esters were in the range of δ 6.18–6.74 [12,14,16–18]. Reported ^13^C NMR chemical shifts for C–O signals of similar 6-membered cyclic nitronic esters were δ 77–87 and for N=C signals were δ 110–126 [14]. Nitronic esters **2–7** exhibit similar signals consistent with the general structural assignment. Stereochemical assignments were made in the following way. The very low amount of nitronic esters **6** and **7** formed from pure *E*-diene in toluene is attributed to minimal crossover. Thus, retention of *cis*-dimethyl stereochemistry at C-3, C-4 has been assumed for **4** and **5** and retention of *trans*-dimethyl stereochemistry from *Z*-diene precursor has been assumed for nitronic esters **6** and **7**. Nitronic esters **5** and **6** exhibit large coupling constants (*J*_4,5_ respectively) consistent with *trans*-diaxial H-4, H-5 stereochemistry. Nitronic ester **2** possesses large coupling constants for one of two H-5 signals (*J*_4,5_ = *J*_5,6_ = 11.2 Hz) that require an axial–axial–axial H-4, H-5, H-6 atom placement. Thus, the phenyl substituent must be located *cis* to the alkenyl substituent in **2** and *trans* in **3**. As confirmation of the assignments, nitronic ester **2** is thermally converted to nitro compound **18** and **3** to **19** by [3,3]-sigmatropic rearrangements (vide infra).

**Table 2:** Characteristic spectral bands for nitronic esters.

Compd.	C=N (cm^−1^)	N=CH (ppm)	N=C (ppm)	C-O (ppm)	*J*_3,4_ (Hz)^a^	*J*_4,5_ (Hz)^a^

**2**	1614	6.38	113.8	86.7		
**3**	1615	6.48	112.7	83		
**4**	1618	6.42	113	87	2.5	6.4
**5**	1619	6.36	114.1	87.3	2.9	10.7
**6**	1620	6.26	114.9	87.7	2.9	11.2
**7**	1615	6.53	113	86.4	2.9	6.6

^a^Atom numbering as shown for nitronic ester **2** (Table 1).

### Sn(IV)-catalyzed reactions of nitronic esters

**Scheme 2:**
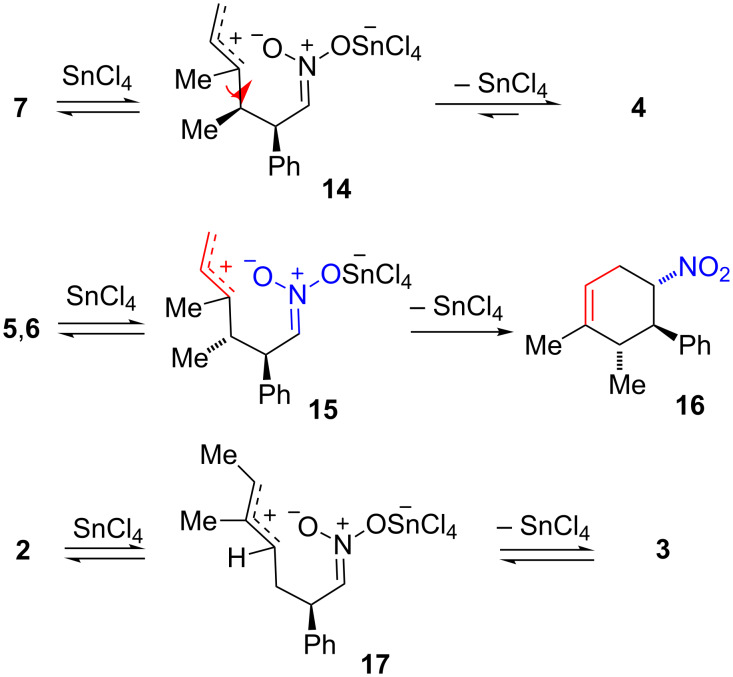
Sn(IV)-catalyzed isomerization of nitronic esters.

The possibility that ternary adduct **12a**,**b** could arise from ring opening of one of the nitronic esters was considered. Neither nitronic ester **4** nor **7** afforded **12a**,**b** when treated with tin(IV) chloride in toluene. That is not to say that the reaction of the nitronic esters with tin(IV) chloride did not occur at all, however. Nitronic ester **7** was converted to nitronic ester **4** (99:1 **7**/**4** final ratio) when it was subjected to tin(IV) chloride for sixteen hours at room temperature (Scheme 2). Similarly, nitronic ester **6** (as a 48:52 mixture with **4**) was partially isomerized to nitronic ester **5** (33:17:50 **6**/**5**/**4** final ratio) when it was subjected to tin(IV) chloride for two hours at room temperature. Nitronic ester **4** did not isomerize under these conditions.

The effect of tin(IV) chloride upon nitronic esters **2** and **3** was also examined. Starting with either nitronic ester **2** or nitronic ester **3** resulted in a final 60:40 ratio of **2**,**3**, respectively, after only 20 minutes. Bond scission to give zwitterions is thought to lead to isomerization in these reactions. These zwitterion intermediates are thought to be characterized by proximate oppositely charged centers (a “cyclo” conformation) as opposed to looser non-proximate oppositely charged centers (an “extended” conformation as shown for **11**). In accordance, it is proposed that tin(IV) complexes nitronic ester **7** and bond scission occurs to give the cyclo zwitterion **14**. Cyclo zwitterion **14** is structurally identical with zwitterion **11a** but remains in a conformation where the opposite charges are in close proximity. Free rotation around the allylic C–C sigma bond of **14** as shown and subsequent reclosure can lead to either regeneration of nitronic ester **7** or formation of nitronic ester **4**, presumably depending on relative thermodynamic stabilities of the two nitronic esters. Thus, it is thought that nitronic ester **4** is at least 2 kcal/mol more stable than nitronic ester **7**. Interconversion of nitronic esters **5** and **6** is thought to occur via the analogous cyclo zwitterion **15**, structurally identical with **11b**. Interestingly, these cyclo zwitterions should be capable of closing in multiple ways, either to give nitronic esters or to give isomeric nitro compounds. Nitronic ester **5** was first converted to a mixture of **5** and **6** and eventually in 15% yield to nitro compound **16** after exposure to tin(IV) chloride for two days. It is quite possible that zwitterion **15** is involved but a larger range of motion over the protracted reaction time allows eventual conversion to the more stable nitro compound. It is also possible that a concerted tin(IV)-catalyzed [3,3]-sigmatropic rearrangement of nitronic ester **5** affords nitro compound **16** (vide infra).

The rapid interconversion of nitronic esters **2** and **3** likely reflects the greater ease of rotation for the cyclo zwitterion **17**. Here bond breaking and reformation is at a secondary rather than tertiary center. A more rapid change of the face presented to the nitronate-O atom would be expected to occur for cyclo zwitterion **17** because top-to-bottom passage past an H-atom should be more rapid than passage past a methyl group as required for the cyclo zwitterions **14** and **15**.

### [3,3]-Sigmatropic rearrangement of nitronic esters

**Scheme 3:**
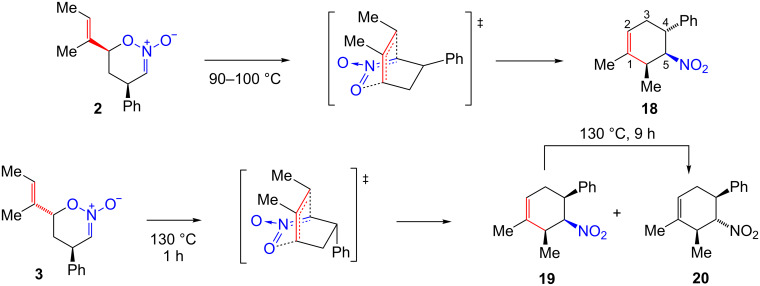
Thermal rearrangement of nitronic esters **2** and **3**.

A variety of *O*-allyl nitronic esters have previously been shown to undergo a non-catalyzed thermal rearrangement to γ,δ-unsaturated nitro compounds [1]. Nitronic esters **2** and **3** also undergo this rearrangement (Scheme 3). Thus, nitro compound **18** is obtained in 74% yield when a DMF solution of nitronic ester **2** is heated at 90–100 °C for one hour. The stereochemical assignment is based on the large (*J*_4,5_ = 12.2 Hz) and small (*J*_5,6_ = 5.8 Hz) ^1^H NMR coupling constants indicating *trans*-nitro to phenyl and *cis-*nitro to methyl substituent placements, respectively. The two-step cycloaddition/sigmatropic rearrangement sequence affords a useful route to this nitro compound. Heating a mixture of β-nitrostyrene and (*E*)-3-methyl-1,3-pentadiene at 170 °C for five hours gives **18** directly, but only as part of an inseparable mixture containing **16**, **20** and at least one other isomer [19]. Thus, tin(IV)-catalyzed nitronic ester cycloaddition and subsequent [3,3]-sigmatropic rearrangement is a superior method to direct thermal Diels–Alder cycloaddition as a means of obtaining pure nitro compound **18**.

In the rearrangement of nitronic ester **3**, more stringent conditions are required and two product isomers are formed. This is thought to be due to the developing unfavorable *cis*-relationship between nitro and phenyl groups in the transition state. Heating **3** at 130 °C for one hour gave a 70:30 mixture of nitro compounds **19** and **20**, respectively, from which **19** could be obtained pure in 48% yield. Longer reaction times at 70–100 °C did afford relatively selective conversion of **3** to nitro compound **19** but the yield was 14% owing to partial decomposition and incomplete rearrangement of the starting material. Nitro compound **19** is the formal Diels–Alder cycloadduct of *cis*-β-nitrostyrene which is not readily available. When nitro compound **19** is heated in DMF at 130 °C for nine hours, it is completely converted to the *trans*-isomer **20**, obtained in 48% yield after chromatography. Here, too, the stereochemical assignment is based on coupling constants. For nitro compound **19**, two small ^1^H NMR coupling constants (*J*_4,5_ = 3.4 Hz; *J*_5,6_ = 5.4 Hz) were observed consistent with *cis*-nitro to phenyl and *cis-*nitro to methyl substituent placement. Nitro compound **20** exhibited two large diaxial ^1^H NMR coupling constants (*J*_4,5_ = 11.7 Hz; *J*_5,6_ = 9.8 Hz) consistent with *trans*-nitro to phenyl and *trans-*nitro to methyl substituent placement.

**Scheme 4:**
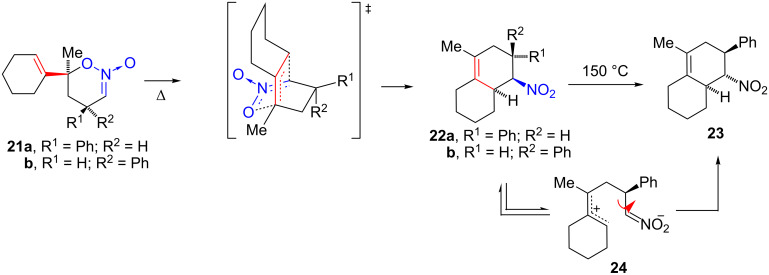
Thermal rearrangement of nitronic esters **21a**, **21b** and **22b**.

These results parallel previously published observations [1] for rearrangement of nitronic esters **21a** and **21b** (Scheme 4). The rearrangement of **21b**, in which the alkenyl and phenyl groups are located *trans* was more difficult, requiring higher temperature than the rearrangement of **21a** (90 °C and 20 °C, respectively). Apparently, the developing *cis*-relationship between the phenyl and nitro groups in transition states leading to both **22b** and **19** is unfavorable. On prolonged heating, product **22b** is converted to the *trans*-isomer **23**. It was postulated that the zwitterion **24** was an intermediate and inversion of the nitro group involved rotation around a C–C single bond. A similar process is likely for conversion of **19** to **20**.

Attempts to thermally rearrange nitronic esters **4**–**7** were unsuccessful. Thus, heating any of these four nitronic esters in the temperature range of 70–150 °C led to decomposition rather than formation of nitro compounds. It is possible that the formation of nitro compound **16** from the mixture of nitronic esters **5** and **6** in the presence of tin(IV) chloride was a catalyzed concerted sigmatropic rearrangement of nitronic ester **5**. However, as previously noted this reaction may well involve zwitterion **15** as the intermediate since nitronic esters **5** and **6** equilibrate under these conditions presumably through **15**.

**Scheme 5:**
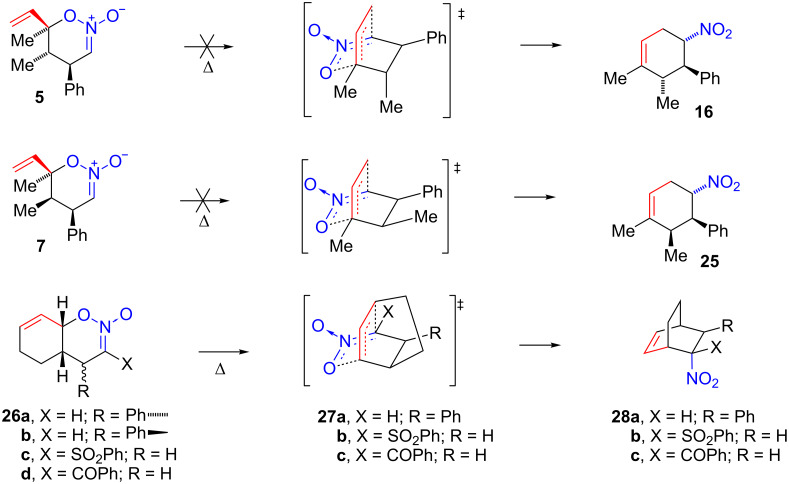
Thermal reactions of nitronic esters **5**, **7**, and **26a–d**.

The thermal stability of nitronic esters **4**–**7** appears to be roughly similar to nitronic esters **2** and **3**: all have half-life’s greater than one hour at 100 °C with the exception of nitronic ester **2** that largely rearranges under these conditions. However, rearrangement of **4**–**7** is too slow to occur prior to decomposition. This is particularly surprising for nitronic ester **5** (Scheme 5). Here the rearrangement should occur to give **16** where the nitro and phenyl groups are *trans* and no other significant steric interactions are apparent for the transition state. Other rearrangements to give products where the nitro and phenyl groups are *trans* (nitro compounds **18** and previously studied **22a**) [1] were successful. Even nitronic ester **26a** affords the bicyclo[2.2.2]alkene **28a** in 72% yield when heated at 150 °C, presumably through strained tricyclic transition state **27a** [1]. In contrast, nitronic ester **26b** fails to undergo rearrangement presumably owing to the requirement that the nitro and phenyl groups be *cis* in the product. The rearrangement of nitronic ester **7** to give nitro compound **25** where nitro and phenyl groups would be *trans* also fails, but here a methyl group is *cis* to phenyl in the product.

**Scheme 6:**
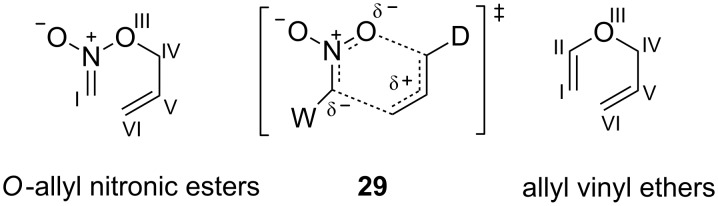
General transition state for the [3,3]-sigmatropic rearrangement of *O*-allyl nitronic esters.

Apparently electron-attracting substituents (W-groups) attached to the nitronic C-atom (the C(I) site, Scheme 6) of *O*-allyl nitronic esters strongly accelerate rearrangement. Nitronic ester **26c** with a sulfone group (Hammett σ*_p_* 0.68) [20] at the C(I) position rearranges at 80 °C to **28b** whereas analogous nitronic ester **26d** with phenacyl group, a less powerful W-group (Hammett σ*_p_* 0.43) [20], requires 120 °C for rearrangement to **28c** [2]. The [3,3]-sigmatropic rearrangement of **26a**, which lacks a W-group at C(I), required especially vigorous conditions (150 °C) [1]. Nitronic ester **26a** possesses a phenyl group not present in either **26c** or **26d**. Thus, the transition state **27a** is not strictly comparable to **27b** or **27c** but should be similar. These results lead to the proposal of dipolar general transition state **29**. Negative charge in transition state **29** should be stabilized by a W-group at C(I). Substantial partial charge development would seem to be present from the observed results. In contrast, a W-group attached at C(I) of an allyl vinyl ether slightly decelerates a Claisen rearrangement [3,21].

Nitronic ester **21a** rearranges more readily than nitronic ester **2** (20 °C vs 70 °C, respectively, 1 day) [1]. Likewise, nitronic ester **21b** rearranges more easily than nitronic ester **3** (90 °C vs 130 °C, respectively, 1–2 hours). All four of these nitronic esters possess a trisubstituted alkene but only nitronic esters **21a** and **21b** possess a methyl group at the C(IV) site. Possibly the rate differences are due to an electronic accelerative effect arising from the electron-donating C(IV) methyl group (D-group) as shown in transition state **29**. It is also true that **21a** and **21b** rearrange to tetrasubstituted cycloalkenes **22a** and **22b**, respectively, whereas **2** and **3** give less stable trisubstituted alkenes **18** and **19** [22] which may explain the rate difference. Significant steric differences between the transition states leading to **22a** and **22b** versus **18** and**19** appear to be absent.

**Scheme 7:**
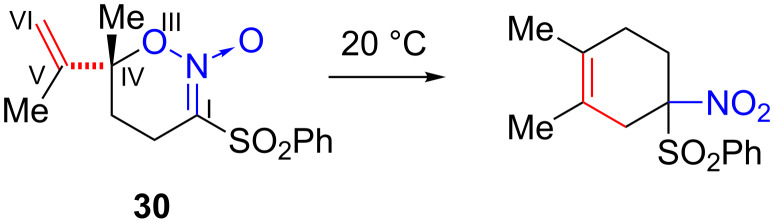
Thermal rearrangement of nitronic ester **30**.

Nitronic esters **4**–**7** lack methyl substituents at C(V) and C(VI) whereas other nitronic esters that do undergo rearrangement possess methyl or alkyl substituents at these positions. The absence of a methyl substituent in **4**–**7** at C(VI) is a likely contributing cause of failed rearrangement. In the Claisen rearrangement, a methyl group has a small accelerative effect and alkoxy groups a larger accelerative effect when located at the C(VI) site of the allyl vinyl ether [3,23]. These observations were attributed to an electronic effect whereby the C(VI)-substituent weakened the O–C(IV) bond and hence the rearrangement energy barrier. A similar effect should operate with *O*-allyl nitronic esters resulting in stabilization of transition state **29** by C(VI) alkyl substituents. It should be noted, however, that the nitronic ester **30** (Scheme 7) also lacks a C(VI) substituent: it undergoes a rearrangement at room temperature and a similar bicyclic transition state must be involved [2]. The high reactivity of nitronic ester **30** is presumably due to the combined presence of a C(I) sulfone group and a C(IV) methyl group. In particular, the presence of an electron-attracting substituent at C(I) appears to have a greater accelerative effect on the rearrangement than the presence of electron-donating substituents at C(IV) or C(VI).

## Conclusion

Cycloaddition to (*E*)-3-methyl-1,3-pentadiene occurred predominantly on the less substituted double bond. However, the diene stereointegrity was compromised. Formation of the ternary adduct **12a**,**b** in 10% yield was a competing reaction in toluene: further studies with additional substrates may lead to a useful new 3-component synthesis of nitro compounds, especially considering the observation that only one diastereomer (mixture of *o,p*-isomers) was observed. Interestingly, reaction with (*Z*)-3-methyl-1,3-pentadiene occurred only at the more substituted double bond affording cycloadduct **7** in 45% yield as the major product. These cycloaddition results are consistent with the presence of zwitterionic intermediates.

Tin(IV)-catalyzed nitronic ester isomerization was a generally observed process. Epimerization occurred at C-6 (the C-atom attached to the nitronate O-atom). An intermediate cyclic zwitterion would seem to be present and is consistent with observed relative rates of isomerization.

The rate (and success) of the thermal [3,3]-sigmatropic rearrangement of *O*-allyl nitronic esters appears to depend on electronic effects. The polar transition state **29** has been proposed and appears to be consistent with the currently available rate data.

## Experimental

### General methods

3-Methyl-1,3-pentadiene (*E*/*Z* mixtures), (*E*)-3-methyl-1,3-pentadiene (*E*/*Z*, 99.7:0.3), tin(IV) chloride, and β-nitrostyrene were purchased commercially and used as received. *N*,*N*-Dimethylformamide was distilled (bp 152–153 °C) and a large forerun discarded to ensure solvent dryness. Ethyl acetate was freshly distilled from anhydrous K_2_CO_3_. All reactions were run under a nitrogen atmosphere. Organic layers were dried (MgSO_4_) and concentrated at reduced pressure. Preparative TLC was performed on 250 μm silica gel plates (Analtech). NMR spectra were taken in CDCl_3_ and recorded on a Varian Inova 500 spectrometer. High-resolution mass spectra (HRMS) were taken on a Waters Autospec Ultima Q spectrometer. Chemical ionization (CI) was performed with CH_4_ as carrier gas unless otherwise noted. IR samples were recorded as attenuated total reflectance (ATR) spectra on a Perkin-Elmer 1600 spectrometer. Melting points (uncorrected) were taken on a Thomas-Hooover apparatus and elemental analyses (within 0.3% of theory) were performed by Robertson Microlit Laboratories.

### Synthesis

**Preparation of *Z*-diene 1b** [13]: A stirred mixture of **1a**,**b** (40 mmol, 63:37, **1a**/**b** ratio by ^1^H NMR) and freshly ground maleic anhydride (3 equiv) warmed to 45–50 °C after 10 min. Stirring was continued at ambient temperature for 24 h. The remaining diene was distilled from the reaction mixture by gradually reducing the pressure to 2 mmHg and was collected in a cold trap (dry ice). A 15 mmol portion of pure (>99.5% by ^1^H NMR) *Z*-diene **1b** was obtained.

**General procedure for carrying out Sn(IV)-catalyzed reactions of β-nitrostyrene and 3-methyl-1,3-pentadiene:** A solution of 3-methyl-1,3-pentadiene (either **1a** or **1b**, 2 mmol) in CH_2_Cl_2_ (2 mL) was added dropwise (syringe pump) over 1 h to a cold (−78 °C) mixture of SnCl_4_ (0.5 equiv), β-nitrostyrene (0.5 equiv), and CH_2_Cl_2_ (10 mL). The resulting cold solution was stirred for 40 min. Dichloromethane (10 mL) and then saturated aqueous NaHCO_3_ (5 mL) were added dropwise over 10 min keeping the temperature below −70 °C. The mixture was allowed to warm and the layers were separated. The aqueous layer was extracted (two 10 mL portions of CH_2_Cl_2_) and the combined organic layers were washed with 50:50 brine/water (10 mL), dried, and concentrated to give the crude product. This was chromatographed on a column of silica gel (elution with hexanes/EtOAc from 90:10 to 0:100).

**General procedure for reaction of β-nitrostyrene and 3-methyl-1,3-pentadiene in toluene solution:** Tin(IV) chloride (2 mmol) was added dropwise over 5 min to a cold (−78 °C) solution of β-nitrostyrene (1 equiv) and 3-methyl-1,3-pentadiene (2 equiv, either pure **1a** or a 70:30 **1a**,**b** mixture) in toluene (20 mL). The resulting cold (<−70 °C) solution was stirred for 75 min. Ethyl acetate (10 mL) and then saturated aqueous NaHCO_3_ solution (5 mL) were added dropwise over 5 min keeping the temperature below −50 °C. The mixture was allowed to warm and was extracted with EtOAc (five 10 mL portions). The combined extracts were washed with saturated NaHCO_3_ solution (five 15 mL portions) and brine (five 10 mL portions), dried, and concentrated to give the crude product which was chromatographed on silica gel (hexanes/EtOAc from 95:5 to 0:100). From the (*E*)-diene, ternary adduct **12a**,**b** (**a**/**b** 66:34 ratio; 0.2 mmol; 10% yield), nitronic ester **2** (0.54 mmol, 27% yield), and nitronic ester **3** (0.58 mmol, 29% yield) were obtained as well as minor amounts of nitronic esters **4–7**.

**General procedure for Tin(IV) chloride-catalyzed isomerization of nitronic esters:** Tin(IV) chloride (0.12 mmol) was added dropwise over 2 min to a solution of nitronic ester (1 equiv) in toluene (5 mL) and the resulting solution was stirred for an appropriate time until further isomerization was not observed (20 min for **2** and **3**; 2 h for **6**; 16 h for **7**). Saturated aqueous NaHCO_3_ solution (5 mL) and EtOAc (10 mL) were added. The organic layer was separated and the aqueous layer was extracted with EtOAc (five 10 mL portions). The combined organic layers were washed with saturated aqueous NaHCO_3_ solution (five 10 mL portions) followed by brine (three 10 mL portions), dried, and concentrated to give the crude product which was analyzed spectroscopically.

**[3,3]-Sigmatropic rearrangement of nitronic ester 2:** A solution of **2** (0.12 mmol) in DMF (10 mL) was heated for 2 h at 90–97 °C. Benzene (20 mL) and EtOAc (20 mL) were added to the cooled solution. The resulting solution was washed with water (twenty 10 mL portions), dried, and concentrated. Preparative TLC (hexanes/EtOAc, 90:10) was performed on the residue to afford 0.09 mmol (74% yield) of pure **18**. The analytical sample was recrystallized from benzene/hexanes: mp 100–100.2 °C; TLC *R*_f_ = 0.87 (hexanes/EtOAc, 90:10); IR: 1553, 1375 cm^−1^ (NO_2_); ^1^H NMR δ 7.4–7.15 (m, 5H), 5.42 (br m, 1H), 5.14 (dd, *J* = 5.8, 12.2 Hz, 1H), 3.46 (ddd [24], *J* = 6.5, 11.4, 11.4 Hz, 1H), 2.83 (m, 1H), 2.48 (m, 1H), 2.21 (m, 1H), 1.80 (s, 3H), 1.11 (d, *J* = 7.3 Hz, 3H); ^13^C NMR δ 141.7, 135.2, 128.8, 127.1, 127.1, 120.4, 90.2, 38.3, 37.6, 34.6, 21.8, 14.2; HRMS–CI (*m*/*z*): [M + H]^+^ calcd for C_14_H_18_NO_2_, 232.1338; found, 232.1338; Elemental analysis (%): Anal. calcd C, 72.72; H, 7.36; N, 6.06; found: C, 72.47; H, 7.11; N, 5.89.

## Supporting Information

File 1 Experimental procedures, characterization of compounds **2–7**, **12**, **13**, **16**, and **18–20**, and crystallographic data for compound **12a**,**b**.

File 2 NMR spectra and signal assignments for compounds **2–7**, **12**,**13**, and **18–20**.
