# Supplementary material for: Sequential Diels–Alder/[3,3]-sigmatropic rearrangement reactions of β-nitrostyrene with 3-methyl-1,3-pentadiene
Source: Beilstein J Org Chem. 2013 Oct 17;9:2137–46. doi: 10.3762/bjoc.9.251 (PMC3817504; doi:10.3762/bjoc.9.251)
Supplement: File 1 — Experimental procedures, characterization of compounds 2–7, 12, 13, 16, and 18–20, and crystallographic data for compound 12a,b. [file Beilstein_J_Org_Chem-09-2137-s001.pdf]

Supporting Information

for

Sequential Diels–Alder/[3,3]-sigmatropic rearrangement reactions of β-nitrostyrene with 3-methyl-1,3-pentadiene

Peter A. Wade*[1], Alma Pipic[1], Matthias Zeller[2] and Panagiota Tsetsakos[1]

Address: [1]Department of Chemistry, Drexel University, Philadelphia, PA 19104, U.S.A. and [2]Department of Chemistry, Youngstown State University, Youngstown, OH, U.S.A.

Email: Peter A. Wade – wadepa@drexel.edu

* Corresponding author

Experimental procedures, characterization of compounds 2–7, 12, 13, 16, and 18–20, and crystallographic data for compound 12a,b

Cycloaddition reactions ………………………………………………………….. S2–S6

Rearrangements ……………………………………………………………….. S6–S9

Crystallographic data for **12a**,**b** …………..…………………………………..… S9–S20

Cycloaddition reactions

Cycloaddition of β-nitrostyrene to (*E*)-3-methyl-1,3-pentadiene in dichloromethane solution: Crude product obtained by the general procedure was chromatographed on silica gel (elution with hexanes/EtOAc from 90:10 to 0:100). Early fractions contained mostly unidentified hydrocarbons. Later fractions contained 0.55 mmol (55% combined yield) of nitronic esters **2**–**5** and **7** in a 29:28:9:11:23 ratio, respectively. Repetitive chromatography (hexanes/EtOAc and CH_2Cl_2/EtOAc to completely separate **2** from **7**) provided purified samples of all nitronic esters. Elution order from the column was **4**, **3**, **7**, **2**, and finally **5**.

Nitronic ester 2: Chromatography afforded a solid that was recrystallized from EtOAc: mp 80–80.2 °C; TLC R_f = 0.27 (70:30 hexanes/EtOAc); IR (ATR) 1614 cm^{-1} (C=N stretch); ^{1}H NMR δ 7.2-7.4 (m, 5H), 6.38 (d, 1H, *J* = 2.4 Hz), 5.70 (q, 1H, *J* = 6.8 Hz), 4.86 (d, 1H, *J* = 11.2 Hz), 3.92 (ddd,[1] 1H, *J* = 2.9, 7.3, 10.9 Hz), 2.23 (br dd, 1H, *J* = 7.3, 13.7 Hz), 2.02 (ddd,[1] 1H, *J* = 11.2, 11.2, 13.7 Hz), 1.70 (s, 3H), 1.66 (d, 3H, *J* = 6.8 Hz); ^{13}C NMR δ 139.9, 131.2, 129.2, 127.8, 127.3, 126.6, 113.8, 86.7, 40.9, 33.0, 13.3, 11.8; HRMS-CI (*m/z)* calcd for $C_{14}H_{18}NO_2$ $[M + H]^+$ 232.1338, found 232.1337. Elemental analysis (%): Anal. calcd C, 72.72; H, 7.36; N, 6.06; found: C, 72.45; H, 7.02; N, 6.09.

Nitronic ester 3: Chromatography afforded a viscous oil: TLC R_f = 0.34 (70:30 hexanes/EtOAc); IR (ATR) 1615 cm^{-1} (C=N stretch); ^{1}H NMR δ 7.2-7.4 (m, 5H), 6.48 (d, 1H, *J* = 4.4 Hz), 5.60 (q, 1H, *J* = 6.3 Hz), 4.72 (apparent d, 1H, *J* = 9.8 Hz), 3.92 (m,

1H), 2.41 (ddd,[1] 1H, J = 7.3, 10.7, 14.2 Hz), 1.93 (m, 1H), 1.64 (s, 3H), 1.62 (d, 3H, J = 6.8 Hz); ^{13}C NMR δ 141.3, 130.8, 129.1, 127.7, 127.6, 125.7, 112.7, 83.0, 38.1, 31.1, 13.2, 12.0; HRMS-CI (*m/z)* calcd for $C_{14}H_{18}NO_2$ $[M + H]^+$ 232.1338, found 232.1333.

Nitronic ester 4: Chromatography afforded a viscous oil that was nearly pure **4** (98:2 ratio of **4/6**): TLC R_f = 0.44 (70:30 hexanes/EtOAc); IR (ATR) 1618 cm^{-1} (C=N); ^{1}H NMR δ 7.1-7.4 (m, 5H), 6.42 (d, 1H, J =2.5 Hz), 5.96 (dd, 1H, J = 10.8, 17.1 Hz), 5.55 (d, 1H, J = 17.1 Hz), 5.34 (d, 1H, J = 10.8 Hz), 4.10 (dd, 1H, J = 2.5, 6.4 Hz), 1.95-2.05 (m, 1H), 1.48 (s, 3H), 0.75 (d, 3H, J =7.3 Hz); ^{13}C NMR δ 139.0, 138.1, 128.8, 128.3, 127.5, 115.4, 113.0, 87.0, 42.9, 36.0, 24.4, 9.6; HRMS-CI (*m/z)* calcd for $C_{14}H_{18}NO_2$ $[M + H]^+$ 232.1338, found 232.1333.

Nitronic ester 5: Chromatography afforded a viscous liquid: IR (ATR) 1619 cm^{-1} (C=N); ^{1}H NMR δ 7.2-7.4 (m, 5H), 6.36 (d, 1H, J = 2.9 Hz), 5.87 (dd, 1H, J = 10.7, 17.6 Hz), 5.47 (d, 1H, J = 17.6 Hz), 5.38 (d, 1H, J = 10.7 Hz), 3.32 (dd, 1H, J = 2.9, 10.7 Hz), 2.01 (m, 1H), 1.56 (s, 3H), 0.88 (d, 3H, J = 6.8 Hz); ^{13}C NMR δ 138.7, 137.8, 129.1, 128.5, 128.0, 117.8, 114.1, 87.3, 46.4, 40.6, 14.8, 13.5; HRMS-CI (*m/z)* calcd for $C_{14}H_{18}NO_2$ $[M + H]^+$ 232.1338, found 232.1331.

Nitronic ester 7: Chromatography afforded a solid. This was recrystallized from benzene/hexanes: mp 105–106 ^{o}C; IR (ATR) 1615 cm^{-1} (C=N); ^{1}H NMR δ 7.2-7.4 (m, 5H), 6.53 (d, 1H, J = 2.9 Hz), 5.83 (dd, 1H, J = 11.2, 17.6 Hz), 5.43 (d, 1H, J = 17.6 Hz), 5.25 (dd, 1H, J = 1, 11.2 Hz), 4.18 (dd, 1H, J = 2.9, 6.6 Hz), 2.05 (m, 1H), 1.65 (s,

3H), 0.70 (d, 3H, *J* = 6.8 Hz); ^{13}C NMR δ 138.1, 137.5, 128.8, 128.7, 127.6, 114.9, 113.0, 86.4, 42.5, 36.8, 23.4, 11.6; HRMS-CI (*m/z)* calcd for $C_{14}H_{18}NO_2$ [M + H]$^+$ 232.1338, found 232.1340.

Cycloaddition of β-nitrostyrene to (*Z*)-3-methyl-1,3-pentadiene in dichloromethane solution: Crude product obtained by the general procedure was chromatographed on silica gel (elution with hexanes/EtOAc from 90:10 to 0:100). Very early fractions contained mostly unidentified hydrocarbons. Compound **13** (0.09 mmol) eluted next followed by 0.61 mmol (61% combined yield) of nitronic esters **4–7** in a 2:6:19:73 ratio, respectively. Elution order was **4** and **6** (only partial separation was possible), **7**, and finally **5**. The sample of **6** was not obtained pure.

Nitronic ester 6: Chromatography afforded a viscous oil that was an 80:12:08 mixture of **6**/**4**/**7**, respectively. IR (ATR) 1620 cm^{-1} (C=N); ^{1}H NMR δ 7.2-7.4 (m, 5H), 6.25 (d, 1H, *J* = 2.9 Hz), 5.98 (dd, 1H, *J* = 11.2, 16.8 Hz), 5.62 (d, 1H, *J* =16.8 Hz), 5.39 (d, 1H, *J* = 11.2 Hz), 3.18 (dd, 1H, *J* = 2.9, 11.2 Hz), 2.03 (m, 1H), 1.44 (s, 3H), 0.94 (d, 3H, *J* = 6.8 Hz); ^{13}C NMR (taken on a 52:48 mixture of **4** and **6**) signals attributed to **6**: δ 138.6, 132.9, 129.0, 128.4, 128.0, 117.3, 114.9, 87.7, 46.3, 41.4, 24.8, 12.8; HRMS-CI (*m/z)* calcd for $C_{14}H_{18}NO_2$ [M + H]$^+$ 232.1338, found 232.1330.

Open-chain adduct 13: Chromatography afforded an oil: IR (ATR) 1656 (C=C),1554, 1379 cm^{-1} (NO_2); ^{1}H NMR δ 7.1-7.3 (m, 5H), 5.33 (t, 1H, *J* = 7.8 Hz), 4.72 (dd, 1H, *J* = 5.0, 12.2 Hz), 4.64 (dd, 1H, *J* = 9.7, 12.2), 3.92 (dd, 1H, *J* = 8.2, 11.7 Hz), 3.87 (dd, 1H,

J = 7.8, 11.7), 3.54 (ddd, 1H, *J* = 5.0, 5.3, 9.7 Hz), 2.55 (m, 1H), 1.56 (s, 3H), 1.15 (d, 3H, *J* = 6.8 Hz); ^{13}C NMR δ 143.1, 138.5, 128.7, 128.0, 127.7, 123.2, 78.6, 47.8, 46.0, 40.3, 16.7, 13.6; negative ion HRMS-CI (*m/z)* calcd for $C_{14}H_{18}NO_2{}^{35}Cl_2$ [M + ^{35}Cl]$^-$ 302.0715, found 302.0720.

Cycloaddition of β-nitrostyrene to 3-methyl-1,3-pentadiene in toluene solution: The crude product was prepared from a 70:30 *E*/*Z*-diene mixture as described in the general procedure and was chromatographed on silica gel (hexanes/EtOAc from 95:5 to 0:100). Early fractions contained mostly unidentified hydrocarbons followed by 0.24 mmol (12% yield) of ternary adduct **12a**,**b** (66:34 **a**/**b** ratio). Latter fractions contained 0.94 mmol (47% combined yield) of nitronic esters **2**–**7** in a 32:40:5:5:10:8 ratio, respectively. Repetitive chromatography provided pure samples of all nitronic esters except for a mixture of **4** and **6**. Elution order from the column was **4**,**6** mixture, **3**, **7**, **2**, and finally **5**.

Ternary adduct 12a,b: Occurred as an inseparable mixture of two isomers. Compound **12a** is the major isomer and is slightly more mobile than **12b**. Early chromatography fractions (95:5 hexanes/EtOAc) contained an enriched sample of **12a** (83:16, **a**/**b** ratio) and latter fractions contained a sample enriched in **12b** (40:60, **a**/**b** ratio). Recrystallization of the fraction enriched in **12a** from ethanol afforded a white solid (81:19, **a**/**b** ratio): mp 63.5–64 °C; IR (ATR) 1553 cm^{-1} and 1371 cm^{-1} (NO_2); ^{1}H NMR [independent signals attributed to **12b** that were reduced in intensity for a sample enriched in **12a**] δ 6.95-7.3 (m, 7H of **12a** and 8H of **12b**), 6.62 (d, 2H of **12a**, *J* = 7.8

Hz), [6.53 (d, 1H of **12b**, *J* = 7.3 Hz)], 5.18 (t, 1H of **12a**, *J* = 7.3 Hz), [5.12 (t, 1H of **12b**, *J* = 6.6 Hz)], 4.73 (dd, 1H of **12a**, *J* = 4.9, 12.2 Hz), 4.60 (dd, 1H of **12a**, *J* = 10.3, 12.2 Hz), [4.59 (dd, 1H of **12b**, *J* = 10.3, 12.6 Hz)], 3.54 (m, 1H), 3.18 (dd, 1H of **12a**, *J* = 8.3, 16.1 Hz), 3.08 (m, 1H of **12a** and 2H of **12b**), 2.56 (m, 1H), 2.28 (s, 3H of **12a**), [2.06 (s, 3H of **12b**)], 1.55 (s, 3H of **12a**), [1.54 (s, 3H of **12b**)], [1.17 (d, 3H of **12b**, *J* = 6.8 Hz)], 1.16 (d, 3H of **12a**, *J* = 7.3 Hz); ^{13}C NMR **12a**,**b** (signals absent from the sample enriched in **12a** and therefore assigned to **12b**) δ 139.0, (138.8), 137.5, (137.1), 137.0, (135.9), 134.9, (129.7), 128.8, 128.4, (128.0), 127.9, (127.8), (127.2), 127.1, 125.8, (125.7), (125.1), (79.1), 78.9, 47.8, 46.2, 33.1, (31.3), 20.8, (19.2), (17.1), 17.0, 12.7, (12.68); HRMS-EI *(m/z)* calcd for $C_{21}H_{25}NO_2$ $[M]^+$ 323.1863, found 323.1885; Elemental analysis (%): Anal. calcd C, 78.02; H, 7.74; N, 4.33; found: C, 78.38; H, 7.85; N, 4.47.

Rearrangements

Tin(IV)-catalyzed rearrangement of nitronic ester 5 to nitro compound 16: Tin(IV) chloride (1.3 equiv) was added dropwise over 2 min to a solution of **5** (0.06 mmol) in toluene (5 mL) at 20 °C and the resultant was stirred for 55 h. Saturated aqueous $NaHCO_3$ solution (5 mL) and EtOAc (10 mL) were added. The organic layer was separated and the aqueous layer was extracted with EtOAc (four 10 mL portions). The combined organic layers were washed with saturated $NaHCO_3$ solution (three 10 mL portions) followed by brine (three 10 mL portions), dried, and concentrated to give 0.05 mmol (85% yield) of crude product. The ^{1}H NMR spectrum showed **16** and no remaining nitronic ester. Preparative TLC (90:10 hexanes/EtOAc) of the crude product afforded 0.01 mmol (15% yield) of pure **16**: mp 74–77 °C; IR (ATR) 1548 cm^{-1}, 1372 cm^{-1} (NO_2);

^{1}H NMR δ 7.2-7.4 (m, 5H), 5.43 (br m, 1H), 4.94 (ddd,[1] 1H, J = 5.4, 11.2, 11.2 Hz), 3.0 (dd, 1H, J = 10.3, 11.7 Hz), 2.76 (m, 1H), 2.66 (m, 1H), 2.41 (m, 1H), 1.74 (s, 3H), 0.93 (d, 3H, J = 7.0 Hz); HRMS-CI (NH_3 carrier) $C_{14}H_{18}N_1O_2$ M+1 found 232.1335, calculated 232.1338. The ^{13}C NMR spectrum was not recorded owing to the limited quantity of sample.

Thermal rearrangement of nitronic ester 2: A DMF (10 mL) solution of **2** (0.12 mmol) was heated for 2 h at 90–97 °C. Benzene (20 mL) and EtOAc (20 mL) were added to the cooled solution. The resulting solution was washed with water (twenty 10 mL portions), dried, and concentrated. Preparative TLC (hexanes/EtOAc, 90:10) was performed on the residue to afford 0.09 mmol (74% yield) of pure **18**. The analytical sample was recrystallized from benzene/hexanes: mp 100–100.2 °C; TLC R_f = 0.87 (hexanes/EtOAc, 90:10); IR (ATR) 1553 cm^{-1}, 1375 cm^{-1} (NO_2); ^{1}H NMR δ 7.15-7.4 (m, 5H), 5.42 (br m, 1H), 5.14 (dd, 1H, J = 5.8, 12.2 Hz), 3.46 (ddd,[1] 1H, J = 6.5, 11.4, 11.4 Hz), 2.83 (m, 1H), 2.48 (m, 1H), 2.21 (m, 1H), 1.80 (s, 3H), 1.11 (d, 3H, J =7.3 Hz); ^{13}C NMR δ 141.7, 135.2, 128.8, 127.1, 127.1, 120.4, 90.2, 38.3, 37.6, 34.6, 21.8, 14.2; HRMS-CI (*m/z)* calcd for $C_{14}H_{18}NO_2$ $[M + H]^+$ 232.1338, found 232.1338; Elemental analysis (%): Anal. calcd C, 72.72; H, 7.36; N, 6.06; found: C, 72.47; H, 7.11; N, 5.89.

Thermal rearrangement of nitronic ester 3: A DMF (10 mL) solution of nitronic ester **3** (0.19 mmol) was heated at 128–130 °C for 1 hour under N_2. The solution was cooled to room temperature, diluted with benzene and EtOAc (10 mL each), and washed with water (twenty 10 mL portions). The organic layer was dried and concentrated. The

crude product was a 70:30 mixture of nitro compounds **19** and **20** respectively, based on the ^{1}H NMR spectrum. Preparative TLC (hexanes/EtOAc, 90:10) gave 0.04 mmol (20% yield) of **20** as a more mobile fraction and 0.09 mmol (48% yield) of **19** as a less mobile fraction. The experiment was repeated except that the reaction solution was heated at 73–75 °C for 22 h. After preparative chromatography, 0.03 mmol (14% yield) of compound **19** was obtained. Nitro compound **20** was not observed and 0.07 mmol of nitronic ester **3** (35% recovery) was isolated.

Nitro compound 19: Chromatography afforded a viscous oil: TLC R_f = 0.81 (hexanes/EtOAc, 90:10); IR (ATR) 1371, 1553 cm^{-1} (NO_2 stretch); ^{1}H NMR δ 7.05-7.4 (m, 5H), 5.69 (br m, 1H), 4.99 (dd, 1H, J = 3.4, 5.4 Hz), 3.26 (ddd,[1] 1H, J = 3.4, 5.4, 11.7 Hz), 2.91 (m, 1H), 2.80 (br m, 1H), 2.30 (m, 1H), 1.75 (s, 3H), 1.13 (d, 3H, J =7.3 Hz); ^{13}C NMR δ 140.1, 131.8, 129.1, 128.0, 127.4, 122.4, 92.9, 43.2, 37.1, 26.6, 21.2, 14.3; HRMS-CI (*m/z)* calcd for $C_{14}H_{18}NO_2$ [M + H]$^+$ 232.1338, found 232.1332.

Nitro compound 20: Chromatography afforded a solid that was recrystallized from aqueous ethanol: mp 71–72°C; TLC R_f = 0.87 (hexanes/EtOAc, 90:10); IR (ATR) 1546, 1372 cm^{-1} (NO_2); ^{1}H NMR δ 7.15-7.35 (m, 5H), 5.52 (br m, 1H), 4.63 (dd, 1H, J = 9.8, 11.7 Hz), 3.35 (ddd,[1] 1H, J = 5.9, 11.7, 11.7 Hz), 2.91 (br m, 1H), 2.25-2.4 (m, 2H), 1.76 (s, 3H), 1.14 (d, 3H, J = 6.8 Hz); ^{13}C NMR δ 139.8, 134.4, 128.7, 127.7, 127.4, 121.1, 96.1, 45.2, 39.9, 33.2, 20.8, 16.1; HRMS-CI (*m/z)* calcd for $C_{14}H_{18}NO_2$ [M + H]$^+$ 232.1338, found 232.1332.

Thermal isomerization of nitro compound 19: A DMF (10 mL) solution of **19** (0.04 mmol) was heated at 125–130 °C for 9 h. The solution was cooled to room temperature, diluted with benzene and EtOAc (15 mL each), and washed with water (twenty 10 mL portions). The organic layer was dried and concentrated at reduced pressure. Preparative TLC (hexanes/EtOAc, 90:10) afforded 0.02 mmol (48% yield) of nitro compound **20**.

Crystallographic description of 12a,b

A selected crystal was mounted with the help of a trace of mineral oil on a twenty micron MITEGEN micromesh mount. Diffraction data were collected on a Bruker AXS SMART APEX CCD diffractometer at 100 K using monochromatic Mo Kα radiation with the omega scan technique. Data for **12a**,**b** were collected, its unit cell determined, and the data integrated and corrected for absorption and other systematic errors using the Apex2 suite of programs [2]. The structure was solved by direct methods and refined by full matrix least squares against F^2 with all reflections using SHELXTL [3]. Non-H atoms were refined anisotropically. Hydrogen atoms were placed in calculated positions and were refined as riding on their respective carrier atoms with Uiso = 1.2 Ueq(C) (1.5 for methyl groups). Experimental details (Table S1), bond lengths, angles, and torsion angles (Table S2) are given. CCDC 829196 contains the supplementary crystallographic data. These data can be obtained free of charge from the Cambridge Crystallographic Data Centre via www.ccdc.cam.ac.uk/data_request/cif.

Crystallographic data indicate that the sample crystal is a solid solution (91.4:8.6, respectively) of the two constitutional isomers **12a**,**b**. One of the two crystallographically independent sites is occupied only by the *p*-methyl isomer **12a** of $C_{21}H_{25}NO_2$ (site A, Figure S1). The other site (site B) is in most cases occupied by molecules of the *p*-isomer **12a**, but in a minority of cases by a molecule of the *o*-isomer **12b** instead. Molecules of **12a** and **12b** differ crystallographically only by the position of a methyl group vs. an H atom [refined occupancy ratio: 0.829(4) to 0.171(4)]. The nitro group (molecule at site B, Figure S2) is in close proximity to the minor occupied methyl group of a neighboring site B molecule (C21B in the X-ray structure numbering scheme) and shows signs of disorder (large thermal ellipsoid of the O-atom, O1B, closest to the methyl group). The disorder was however not modeled.

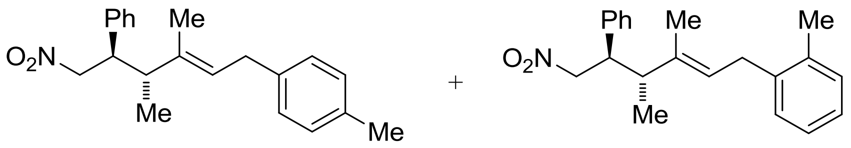

12a 91.4 : 8.6 **12b**

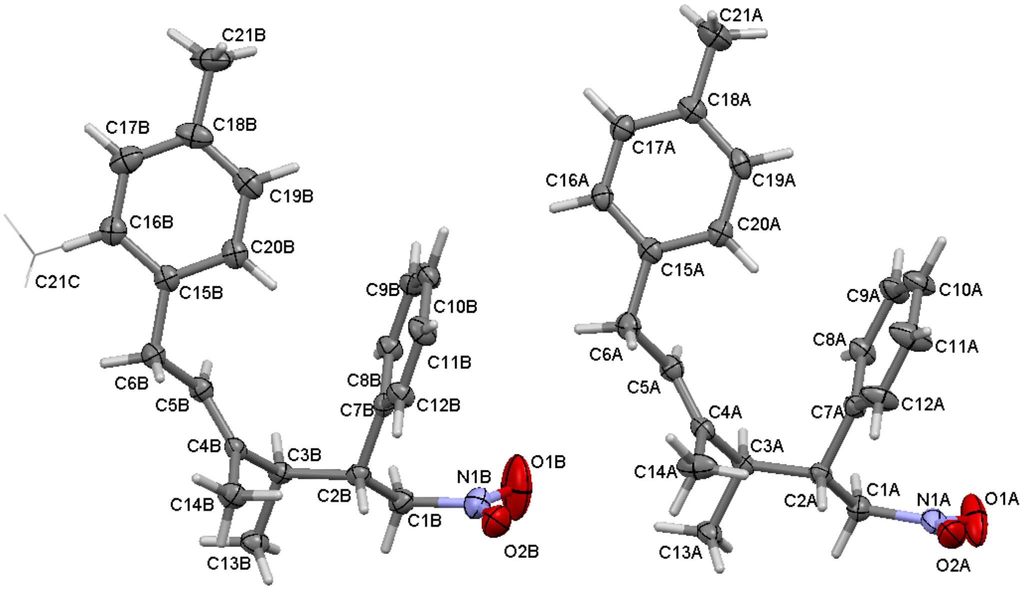

Molecule at site B Molecule at site A (isomer **12a** only)

Figure S1: Asymmetric unit of the structure of compound **12a**,**b** in the solid state with the atom numbering scheme used in the X-ray structure refinement showing the two crystallographically independent molecules A and B. Thermal ellipsoids for non-hydrogen atoms are at the 50% probability level, H atoms are represented as capped sticks. Disorder between molecules **12a** and **12**b is present at site B. The less occupied methyl group, C21C, is shown in wireframe mode.

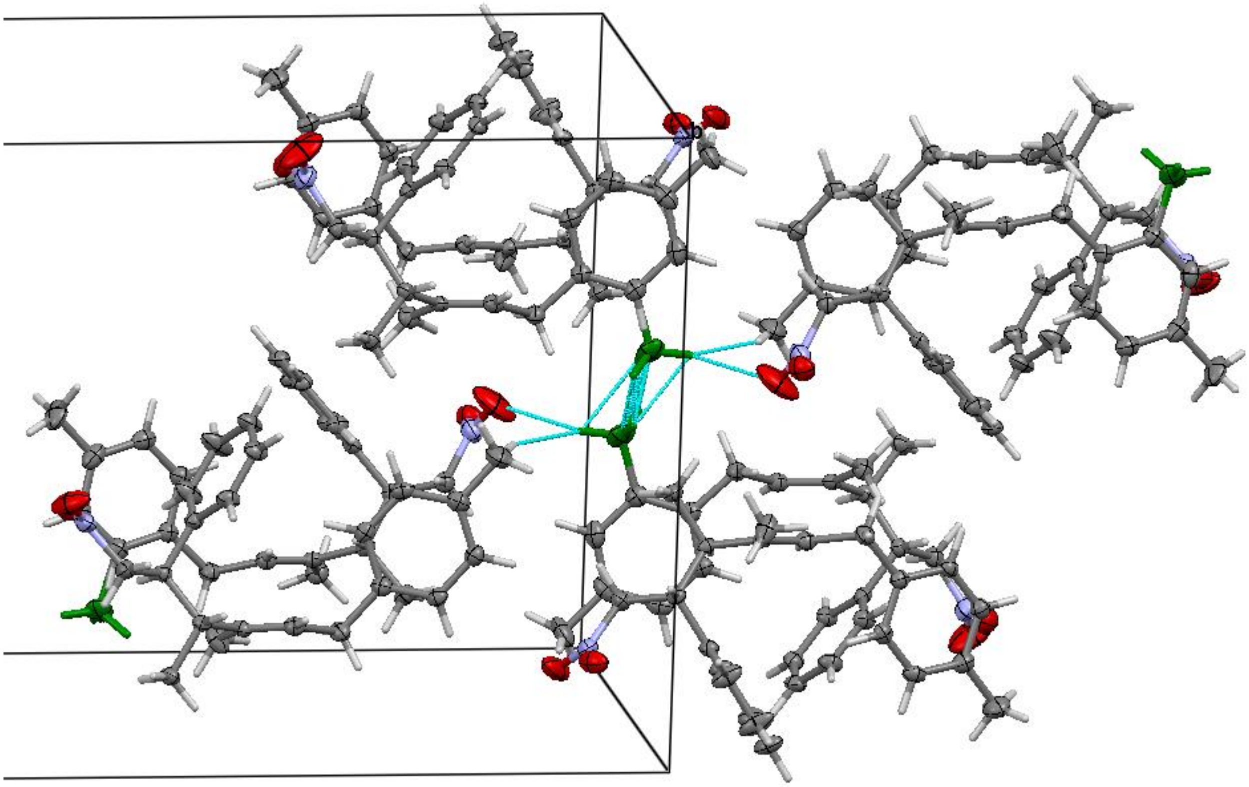

Figure S2: Partial packing diagram showing close contacts between the minor occupied methyl groups of **12b** [highlighted in green; occupancy rate: 17.1(4)%]. Blue lines indicate close contacts between mutually exclusive partially occupied methyl groups, and between each of these methyl groups and the nitro group of a neighboring molecule.

Table S1: Experimental details[a] for obtaining crystallographic data on nitro compound **12a**,**b**.

Crystal data	
Chemical formula	$C_{21}H_{25}NO_2$
M_r	323.42
Crystal system, space group	Monoclinic, $P2_1/n$
Temperature (K)	100
a, *b*, *c* (Å)	9.6634 (18), 24.337 (5), 15.831 (3)
β (°)	98.145 (3)
V (Å^3)	3685.5 (12)
Z	8
F(000)	1392
D_x (Mg m^{-3})	1.166
Radiation type	Mo *K*α
μ (mm^{-1})	0.07
Crystal shape	Plate
Colour	Colourless
Crystal size (mm)	0.37 × 0.18 × 0.06
Data collection	
Diffractometer	Bruker AXS *SMART APEX* CCD diffractometer
Radiation source	fine-focus sealed tube
Monochromator	graphite
Scan method	ω scans
Absorption correction	Multi-scan Apex2 v2009.7-0 (Bruker, 2009)
T_{min}, T_{max}	0.654, 0.746
No. of measured, independent and observed [$I > 2\sigma(I)$] reflections	36933, 9136, 5617
R_{int}	0.067
θ values (°)	θ_{max} = 28.3, θ_{min} = 1.6

Range of *h*, *k*, *l*	*h* = -12→12, *k* = -32→32, *l* = -20→21
Refinement	
$R[F^2 > 2\sigma(F^2)]$, $wR(F^2)$, *S*	0.056, 0.145, 1.02
No. of reflections	9136
No. of parameters	450
No. of restraints	0
H-atom treatment	H-atom parameters constrained
$\Delta\rho_{max}$, $\Delta\rho_{min}$ (e Å^{-3})	0.36, -0.28

[a] Computer programs: Apex2 v2009.7-0 (Bruker, 2009), Apex2 v2009.7-0, *SHELXTL* 6.14 (Bruker, 2000-2003; Sheldrick, 2008), *SHELXTL* 6.14.

Table S2: Selected geometric parameters (Å, º) for nitro compound **12a**,**b**

C1A-N1A	1.501 (2)	C2B-C3B	1.563 (2)
C1A-C2A	1.522 (3)	C2B-H2B	1.0000
C1A-H1A1	0.9900	C3B-C4B	1.518 (2)
C1A-H1A2	0.9900	C3B-C13B	1.538 (2)
C2A-C7A	1.519 (2)	C3B-H3B	1.0000
C2A-C3A	1.558 (2)	C4B-C5B	1.326 (3)
C2A-H2A	1.0000	C4B-C14B	1.513 (2)
C3A-C4A	1.514 (2)	C5B-C6B	1.506 (3)
C3A-C13A	1.539 (2)	C5B-H5B	0.9500
C3A-H3A	1.0000	C6B-C15B	1.511 (3)
C4A-C5A	1.323 (3)	C6B-H6B1	0.9900
C4A-C14A	1.508 (3)	C6B-H6B2	0.9900
C5A-C6A	1.506 (3)	C7B-C12B	1.390 (2)
C5A-H5A	0.9500	C7B-C8B	1.397 (2)
C6A-C15A	1.514 (3)	C8B-C9B	1.383 (3)
C6A-H6A1	0.9900	C8B-H8B	0.9500
C6A-H6A2	0.9900	C9B-C10B	1.383 (3)
C7A-C12A	1.385 (3)	C9B-H9B	0.9500
C7A-C8A	1.389 (2)	C10B-C11B	1.383 (3)

C8A-C9A	1.384 (3)	C10B-H10B	0.9500
C8A-H8A	0.9500	C11B-C12B	1.386 (3)
C9A-C10A	1.377 (3)	C11B-H11B	0.9500
C9A-H9A	0.9500	C12B-H12B	0.9500
C10A-C11A	1.372 (3)	C13B-H13D	0.9800
C10A-H10A	0.9500	C13B-H13E	0.9800
C11A-C12A	1.385 (3)	C13B-H13F	0.9800
C11A-H11A	0.9500	C14B-H14D	0.9800
C12A-H12A	0.9500	C14B-H14E	0.9800
C13A-H13A	0.9800	C14B-H14F	0.9800
C13A-H13B	0.9800	C15B-C16B	1.390 (3)
C13A-H13C	0.9800	C15B-C20B	1.395 (3)
C14A-H14A	0.9800	C16B-C17B	1.389 (3)
C14A-H14B	0.9800	C16B-C21C	1.581 (13)
C14A-H14C	0.9800	C16B-H16B	0.9500
C15A-C16A	1.391 (3)	C17B-C18B	1.393 (3)
C15A-C20A	1.395 (3)	C17B-H17B	0.9500
C16A-C17A	1.383 (3)	C18B-C19B	1.382 (3)
C16A-H16A	0.9500	C18B-C21B	1.492 (3)
C17A-C18A	1.389 (3)	C18B-H18B	0.9500
C17A-H17A	0.9500	C19B-C20B	1.381 (3)
C18A-C19A	1.396 (3)	C19B-H19B	0.9500
C18A-C21A	1.502 (3)	C20B-H20B	0.9500
C19A-C20A	1.385 (3)	C21B-H21D	0.9800
C19A-H19A	0.9500	C21B-H21E	0.9800
C20A-H20A	0.9500	C21B-H21F	0.9800
C21A-H21A	0.9800	C21C-H21G	0.9800
C21A-H21B	0.9800	C21C-H21H	0.9800
C21A-H21C	0.9800	C21C-H21I	0.9800
C1B-N1B	1.498 (3)	N1B-O1B	1.219 (2)
C1B-C2B	1.527 (2)	N1B-O2B	1.221 (2)
C1B-H1B1	0.9900	N1A-O1A	1.214 (2)
C1B-H1B2	0.9900	N1A-O2A	1.228 (2)

C2B-C7B	1.519 (2)		
N1A-C1A-C2A	113.12 (15)	C7B-C2B-C3B	111.27 (14)
N1A-C1A-H1A1	109.0	C1B-C2B-C3B	109.22 (14)
C2A-C1A-H1A1	109.0	C7B-C2B-H2B	108.2
N1A-C1A-H1A2	109.0	C1B-C2B-H2B	108.2
C2A-C1A-H1A2	109.0	C3B-C2B-H2B	108.2
H1A1-C1A-H1A2	107.8	C4B-C3B-C13B	111.41 (14)
C7A-C2A-C1A	111.73 (14)	C4B-C3B-C2B	111.14 (14)
C7A-C2A-C3A	112.35 (14)	C13B-C3B-C2B	112.35 (14)
C1A-C2A-C3A	108.54 (14)	C4B-C3B-H3B	107.2
C7A-C2A-H2A	108.0	C13B-C3B-H3B	107.2
C1A-C2A-H2A	108.0	C2B-C3B-H3B	107.2
C3A-C2A-H2A	108.0	C5B-C4B-C14B	123.70 (17)
C4A-C3A-C13A	110.85 (14)	C5B-C4B-C3B	119.37 (16)
C4A-C3A-C2A	111.33 (14)	C14B-C4B-C3B	116.91 (15)
C13A-C3A-C2A	111.87 (14)	C4B-C5B-C6B	128.91 (17)
C4A-C3A-H3A	107.5	C4B-C5B-H5B	115.5
C13A-C3A-H3A	107.5	C6B-C5B-H5B	115.5
C2A-C3A-H3A	107.5	C5B-C6B-C15B	112.22 (15)
C5A-C4A-C14A	123.23 (17)	C5B-C6B-H6B1	109.2
C5A-C4A-C3A	119.99 (16)	C15B-C6B-H6B1	109.2
C14A-C4A-C3A	116.73 (16)	C5B-C6B-H6B2	109.2
C4A-C5A-C6A	128.08 (17)	C15B-C6B-H6B2	109.2
C4A-C5A-H5A	116.0	H6B1-C6B-H6B2	107.9
C6A-C5A-H5A	116.0	C12B-C7B-C8B	118.11 (16)
C5A-C6A-C15A	111.83 (15)	C12B-C7B-C2B	120.39 (15)
C5A-C6A-H6A1	109.3	C8B-C7B-C2B	121.50 (15)
C15A-C6A-H6A1	109.3	C9B-C8B-C7B	120.93 (17)
C5A-C6A-H6A2	109.3	C9B-C8B-H8B	119.5
C15A-C6A-H6A2	109.3	C7B-C8B-H8B	119.5
H6A1-C6A-H6A2	107.9	C8B-C9B-C10B	120.13 (17)
C12A-C7A-C8A	117.83 (17)	C8B-C9B-H9B	119.9

C12A-C7A-C2A	120.53 (16)	C10B-C9B-H9B	119.9
C8A-C7A-C2A	121.64 (16)	C11B-C10B-C9B	119.73 (18)
C9A-C8A-C7A	120.92 (17)	C11B-C10B-H10B	120.1
C9A-C8A-H8A	119.5	C9B-C10B-H10B	120.1
C7A-C8A-H8A	119.5	C10B-C11B-C12B	120.04 (18)
C10A-C9A-C8A	120.59 (17)	C10B-C11B-H11B	120.0
C10A-C9A-H9A	119.7	C12B-C11B-H11B	120.0
C8A-C9A-H9A	119.7	C11B-C12B-C7B	121.06 (17)
C11A-C10A-C9A	118.95 (19)	C11B-C12B-H12B	119.5
C11A-C10A-H10A	120.5	C7B-C12B-H12B	119.5
C9A-C10A-H10A	120.5	C3B-C13B-H13D	109.5
C10A-C11A-C12A	120.74 (19)	C3B-C13B-H13E	109.5
C10A-C11A-H11A	119.6	H13D-C13B-H13E	109.5
C12A-C11A-H11A	119.6	C3B-C13B-H13F	109.5
C7A-C12A-C11A	120.96 (18)	H13D-C13B-H13F	109.5
C7A-C12A-H12A	119.5	H13E-C13B-H13F	109.5
C11A-C12A-H12A	119.5	C4B-C14B-H14D	109.5
C3A-C13A-H13A	109.5	C4B-C14B-H14E	109.5
C3A-C13A-H13B	109.5	H14D-C14B-H14E	109.5
H13A-C13A-H13B	109.5	C4B-C14B-H14F	109.5
C3A-C13A-H13C	109.5	H14D-C14B-H14F	109.5
H13A-C13A-H13C	109.5	H14E-C14B-H14F	109.5
H13B-C13A-H13C	109.5	C16B-C15B-C20B	118.24 (18)
C4A-C14A-H14A	109.5	C16B-C15B-C6B	121.70 (17)
C4A-C14A-H14B	109.5	C20B-C15B-C6B	120.04 (17)
H14A-C14A-H14B	109.5	C17B-C16B-C15B	120.62 (19)
C4A-C14A-H14C	109.5	C17B-C16B-C21C	117.8 (6)
H14A-C14A-H14C	109.5	C15B-C16B-C21C	121.4 (6)
H14B-C14A-H14C	109.5	C17B-C16B-H16B	119.7
C16A-C15A-C20A	117.90 (17)	C15B-C16B-H16B	119.7
C16A-C15A-C6A	121.51 (16)	C16B-C17B-C18B	120.85 (19)
C20A-C15A-C6A	120.59 (17)	C16B-C17B-H17B	119.6
C17A-C16A-C15A	121.05 (17)	C18B-C17B-H17B	119.6

C17A-C16A-H16A	119.5	C19B-C18B-C17B	118.27 (19)
C15A-C16A-H16A	119.5	C19B-C18B-C21B	118.9 (2)
C16A-C17A-C18A	121.45 (18)	C17B-C18B-C21B	122.8 (2)
C16A-C17A-H17A	119.3	C19B-C18B-H18B	120.9
C18A-C17A-H17A	119.3	C17B-C18B-H18B	120.9
C17A-C18A-C19A	117.46 (18)	C20B-C19B-C18B	121.25 (19)
C17A-C18A-C21A	121.52 (19)	C20B-C19B-H19B	119.4
C19A-C18A-C21A	121.01 (18)	C18B-C19B-H19B	119.4
C20A-C19A-C18A	121.38 (17)	C19B-C20B-C15B	120.76 (19)
C20A-C19A-H19A	119.3	C19B-C20B-H20B	119.6
C18A-C19A-H19A	119.3	C15B-C20B-H20B	119.6
C19A-C20A-C15A	120.77 (18)	C18B-C21B-H21D	109.5
C19A-C20A-H20A	119.6	C18B-C21B-H21E	109.5
C15A-C20A-H20A	119.6	C18B-C21B-H21F	109.5
C18A-C21A-H21A	109.5	C16B-C21C-H21G	109.5
C18A-C21A-H21B	109.5	C16B-C21C-H21H	109.5
H21A-C21A-H21B	109.5	H21G-C21C-H21H	109.5
C18A-C21A-H21C	109.5	C16B-C21C-H21I	109.5
H21A-C21A-H21C	109.5	H21G-C21C-H21I	109.5
H21B-C21A-H21C	109.5	H21H-C21C-H21I	109.5
N1B-C1B-C2B	112.45 (15)	O1B-N1B-O2B	124.0 (2)
N1B-C1B-H1B1	109.1	O1B-N1B-C1B	116.96 (19)
C2B-C1B-H1B1	109.1	O2B-N1B-C1B	119.03 (17)
N1B-C1B-H1B2	109.1	O1A-N1A-O2A	123.63 (19)
C2B-C1B-H1B2	109.1	O1A-N1A-C1A	117.25 (19)
H1B1-C1B-H1B2	107.8	O2A-N1A-C1A	119.08 (17)
C7B-C2B-C1B	111.68 (14)		
N1A-C1A-C2A-C7A	56.1 (2)	C7B-C2B-C3B-C13B	176.89 (14)
N1A-C1A-C2A-C3A	-179.47 (15)	C1B-C2B-C3B-C13B	53.14 (19)
C7A-C2A-C3A-C4A	-54.65 (18)	C13B-C3B-C4B-	-117.82 (18)

		C5B	
C1A-C2A-C3A-C4A	-178.72 (14)	C2B-C3B-C4B-C5B	116.06 (18)
C7A-C2A-C3A-C13A	-179.29 (14)	C13B-C3B-C4B-C14B	61.0 (2)
C1A-C2A-C3A-C13A	56.64 (18)	C2B-C3B-C4B-C14B	-65.11 (19)
C13A-C3A-C4A-C5A	-114.47 (19)	C14B-C4B-C5B-C6B	0.7 (3)
C2A-C3A-C4A-C5A	120.31 (18)	C3B-C4B-C5B-C6B	179.45 (17)
C13A-C3A-C4A-C14A	63.1 (2)	C4B-C5B-C6B-C15B	135.6 (2)
C2A-C3A-C4A-C14A	-62.1 (2)	C1B-C2B-C7B-C12B	-126.69 (18)
C14A-C4A-C5A-C6A	1.6 (3)	C3B-C2B-C7B-C12B	110.97 (18)
C3A-C4A-C5A-C6A	179.03 (17)	C1B-C2B-C7B-C8B	54.2 (2)
C4A-C5A-C6A-C15A	131.2 (2)	C3B-C2B-C7B-C8B	-68.1 (2)
C1A-C2A-C7A-C12A	-120.5 (2)	C12B-C7B-C8B-C9B	0.3 (3)
C3A-C2A-C7A-C12A	117.20 (19)	C2B-C7B-C8B-C9B	179.44 (16)
C1A-C2A-C7A-C8A	59.6 (2)	C7B-C8B-C9B-C10B	-0.4 (3)
C3A-C2A-C7A-C8A	-62.7 (2)	C8B-C9B-C10B-C11B	0.0 (3)
C12A-C7A-C8A-C9A	0.8 (3)	C9B-C10B-C11B-C12B	0.5 (3)
C2A-C7A-C8A-C9A	-179.38 (17)	C10B-C11B-C12B-C7B	-0.6 (3)
C7A-C8A-C9A-C10A	-0.1 (3)	C8B-C7B-C12B-C11B	0.2 (3)
C8A-C9A-C10A-C11A	-1.0 (3)	C2B-C7B-C12B-C11B	-178.93 (17)
C9A-C10A-C11A-C12A	1.4 (4)	C5B-C6B-C15B-C16B	136.37 (18)
C8A-C7A-C12A-C11A	-0.3 (3)	C5B-C6B-C15B-C20B	-42.1 (2)

C2A-C7A-C12A-C11A	179.8 (2)	C20B-C15B-C16B-C17B	-0.2 (3)
C10A-C11A-C12A-C7A	-0.7 (4)	C6B-C15B-C16B-C17B	-178.74 (17)
C5A-C6A-C15A-C16A	134.42 (18)	C20B-C15B-C16B-C21C	-175.3 (6)
C5A-C6A-C15A-C20A	-45.1 (2)	C6B-C15B-C16B-C21C	6.2 (6)
C20A-C15A-C16A-C17A	0.4 (3)	C15B-C16B-C17B-C18B	0.6 (3)
C6A-C15A-C16A-C17A	-179.10 (17)	C21C-C16B-C17B-C18B	175.8 (6)
C15A-C16A-C17A-C18A	0.2 (3)	C16B-C17B-C18B-C19B	-0.8 (3)
C16A-C17A-C18A-C19A	-0.8 (3)	C16B-C17B-C18B-C21B	178.3 (2)
C16A-C17A-C18A-C21A	178.21 (19)	C17B-C18B-C19B-C20B	0.7 (3)
C17A-C18A-C19A-C20A	0.7 (3)	C21B-C18B-C19B-C20B	-178.4 (2)
C21A-C18A-C19A-C20A	-178.28 (18)	C18B-C19B-C20B-C15B	-0.4 (3)
C18A-C19A-C20A-C15A	-0.1 (3)	C16B-C15B-C20B-C19B	0.1 (3)
C16A-C15A-C20A-C19A	-0.5 (3)	C6B-C15B-C20B-C19B	178.67 (17)
C6A-C15A-C20A-C19A	179.05 (16)	C2B-C1B-N1B-O1B	-142.7 (2)
N1B-C1B-C2B-C7B	54.8 (2)	C2B-C1B-N1B-O2B	38.4 (2)
N1B-C1B-C2B-C3B	178.29 (14)	C2A-C1A-N1A-O1A	-150.09 (18)
C7B-C2B-C3B-C4B	-57.52 (18)	C2A-C1A-N1A-O2A	31.9 (2)
C1B-C2B-C3B-C4B	178.74 (14)		

References

1. Analyzed following a reported protocol: Hoye, T. R.; Hanson, P. R.; Vyvyan, J. R. *J. Org. Chem.* **1994**, *59*, 4096-4103.
2. Apex2 v2009.7-0 (2009) Bruker Advanced X-ray Solutions, Bruker AXS Inc., Madison, WI, USA.
3. *SHELXTL* (Version 6.14) (2000-2003) Bruker Advanced X-ray Solutions, Bruker AXS Inc., Madison, WI. USA.
