# Supplementary material for: Sequential Diels–Alder/[3,3]-sigmatropic rearrangement reactions of β-nitrostyrene with 3-methyl-1,3-pentadiene
Source: Beilstein J Org Chem. 2013 Oct 17;9:2137–46. doi: 10.3762/bjoc.9.251 (PMC3817504; doi:10.3762/bjoc.9.251)

Supporting Information

for

Sequential Diels–Alder/[3,3]-sigmatropic rearrangement reactions of β-nitrostyrene with 3-methyl-1,3-pentadiene

Peter A. Wade*[1], Alma Pipic[1], Matthias Zeller[2] and Panagiota Tsetsakos[1]

Address: [1]Department of Chemistry, Drexel University, Philadelphia, PA 19104, U.S.A. and [2]Department of Chemistry, Youngstown State University, Youngstown, OH, U.S.A.

Email: Peter A. Wade – wadepa@drexel.edu

* Corresponding author

NMR spectra and signal assignments for compounds 2–7, 12, 13, 16, and 18–20

^{1}H NMR spectra and signal assignments for **2–7**, **12**, **13**, **16**, **18–20** ……… S2–S14

^{13}C NMR spectra and signal assignments for **2–7**, **12**, **13**, **18–20** ………… S15–S26

DEPT spectra for **2**, **4**, and **12** ……………………………………………… S27–S29

^{1}H NMR Spectrum

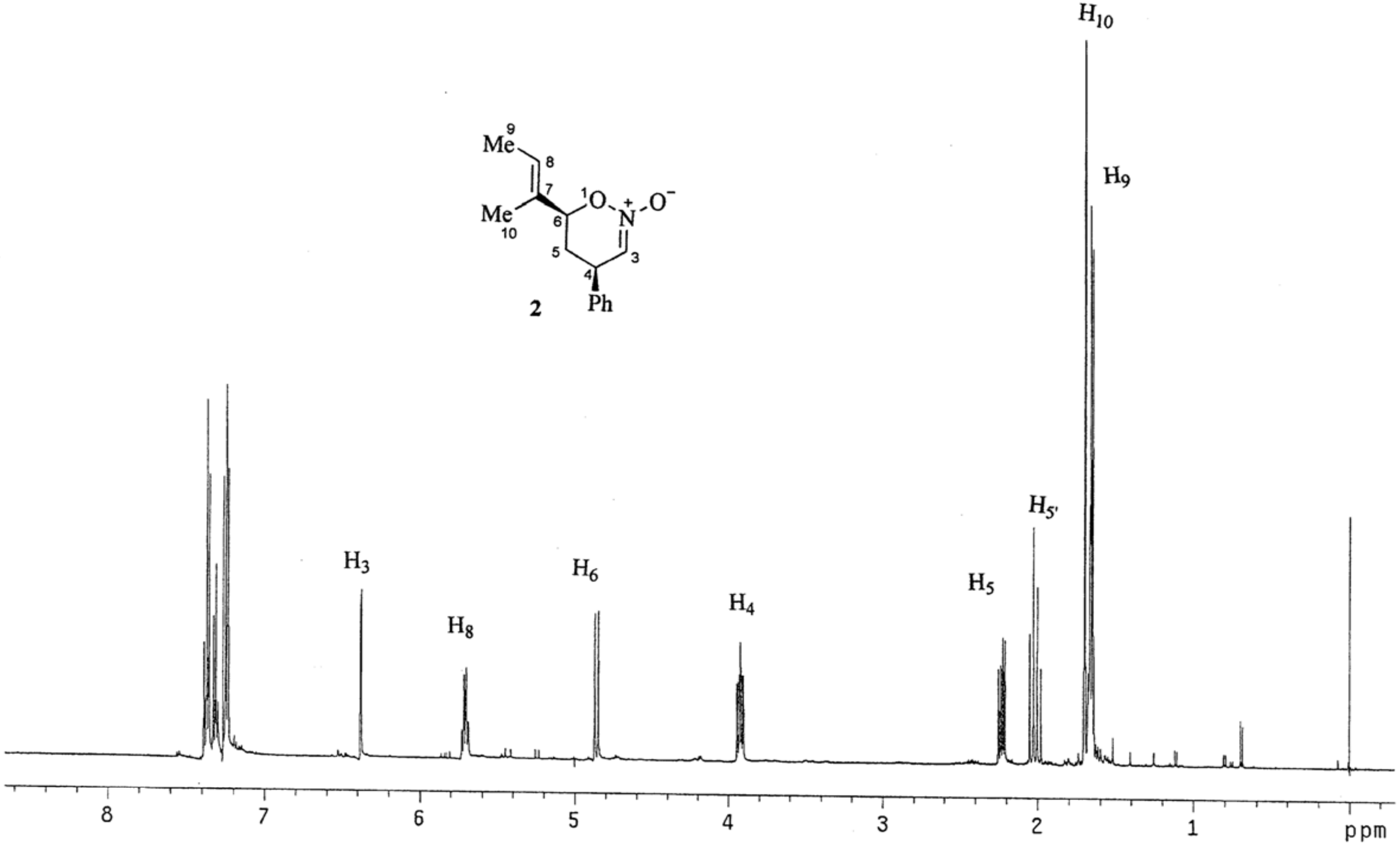

^{1}H NMR Spectrum

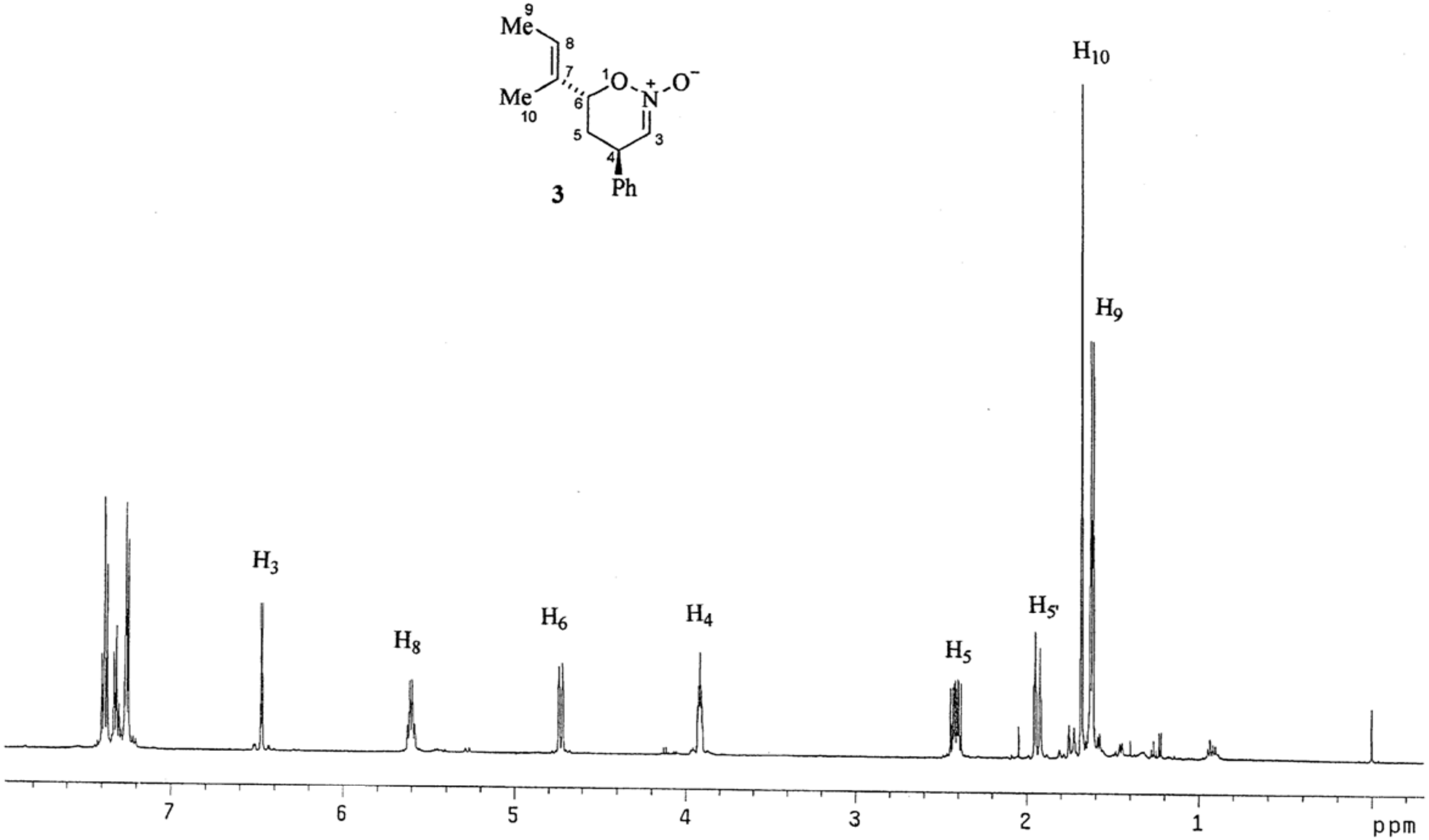

^1H NMR Spectrum

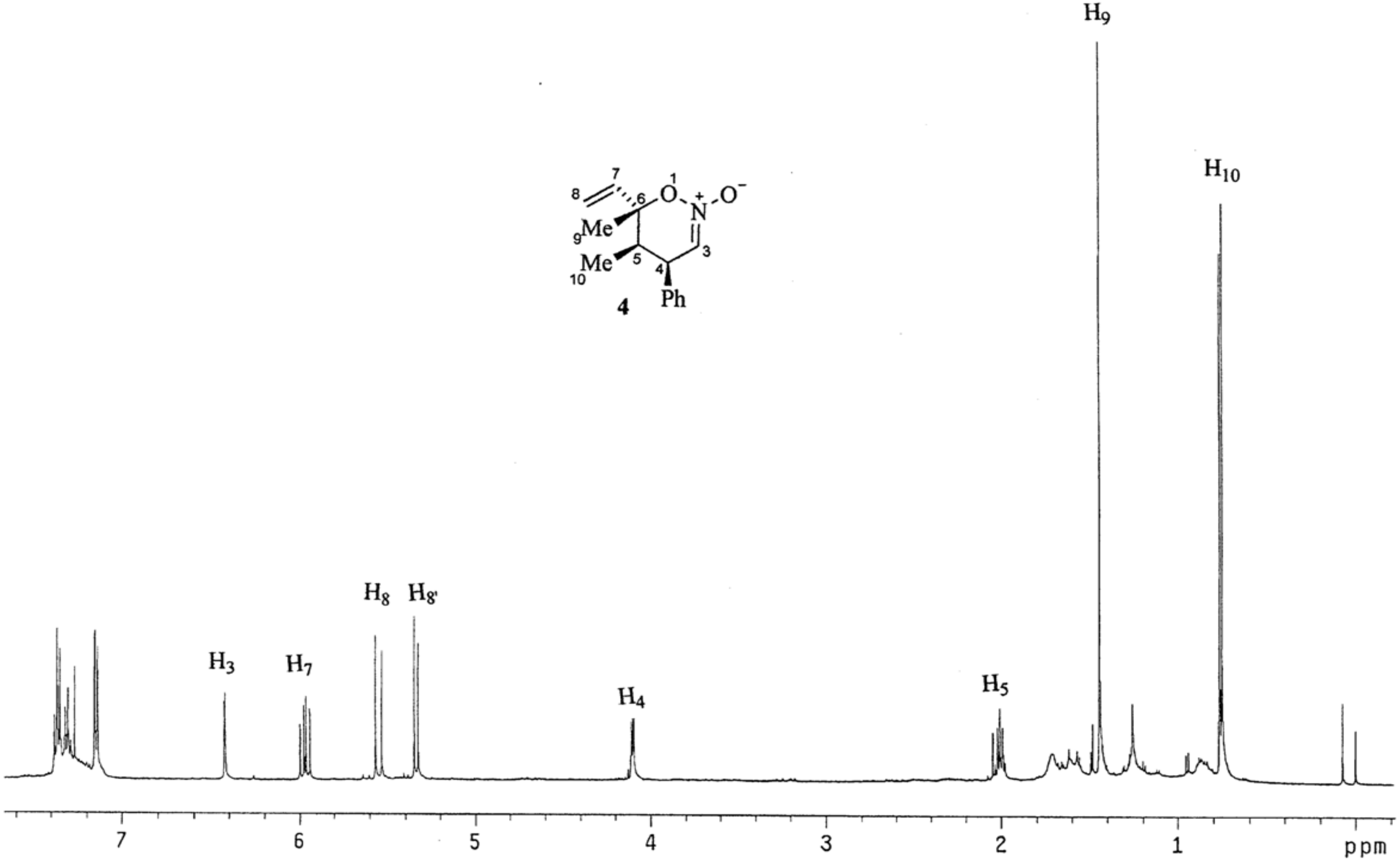

^{1}H NMR Spectrum

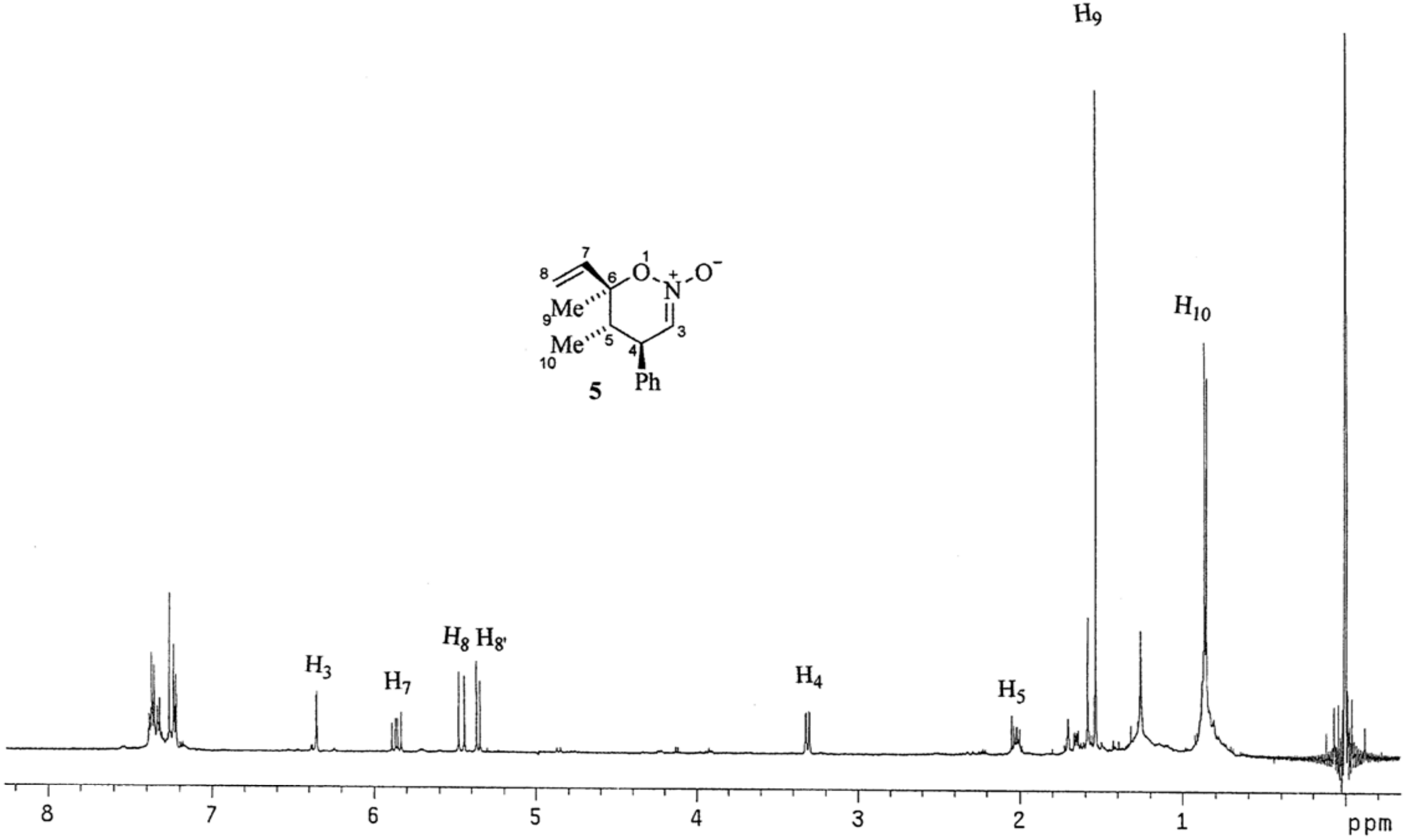

^{1}H NMR Spectrum

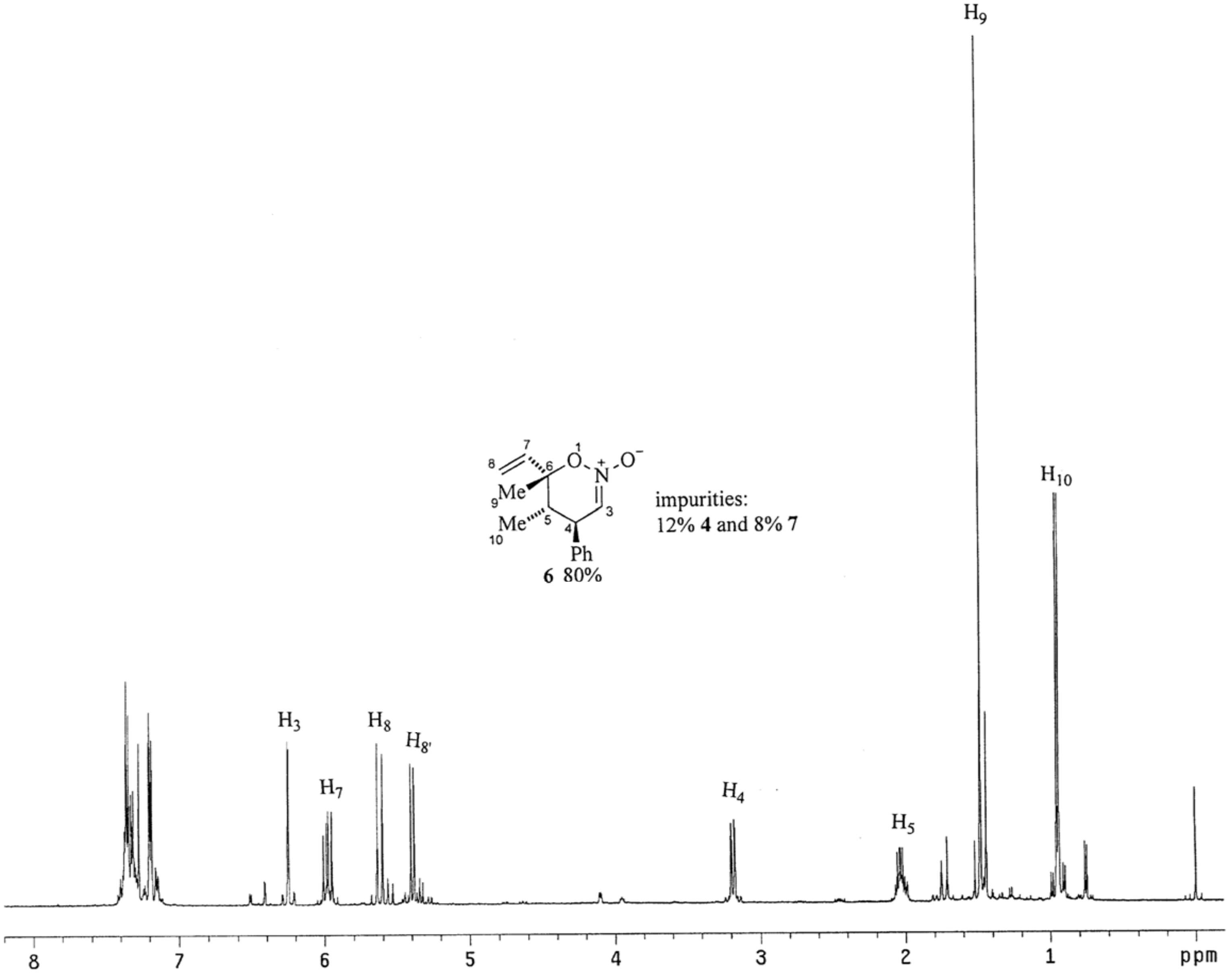

^{1}H NMR Spectrum

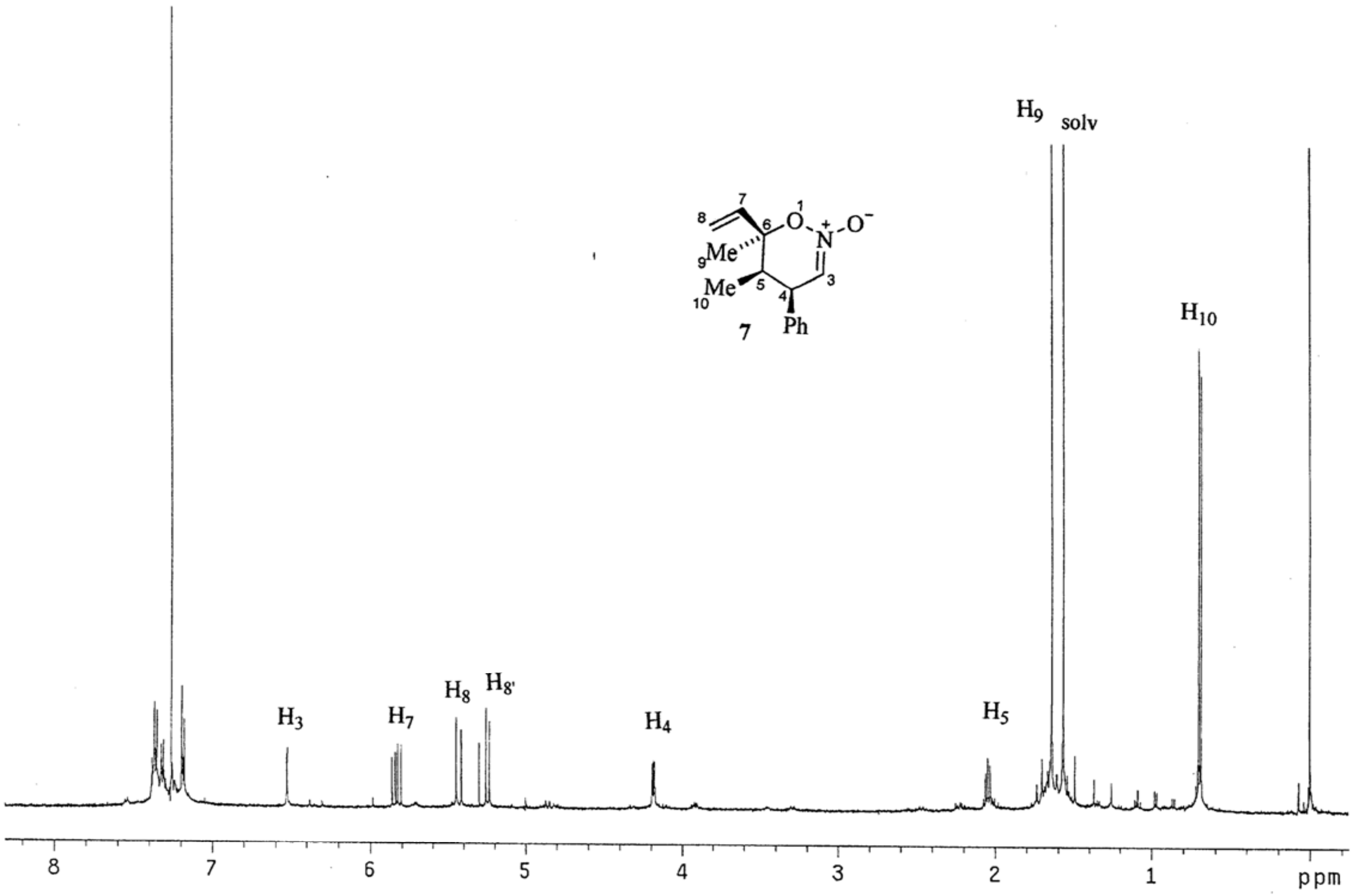

^{1}H NMR Spectrum

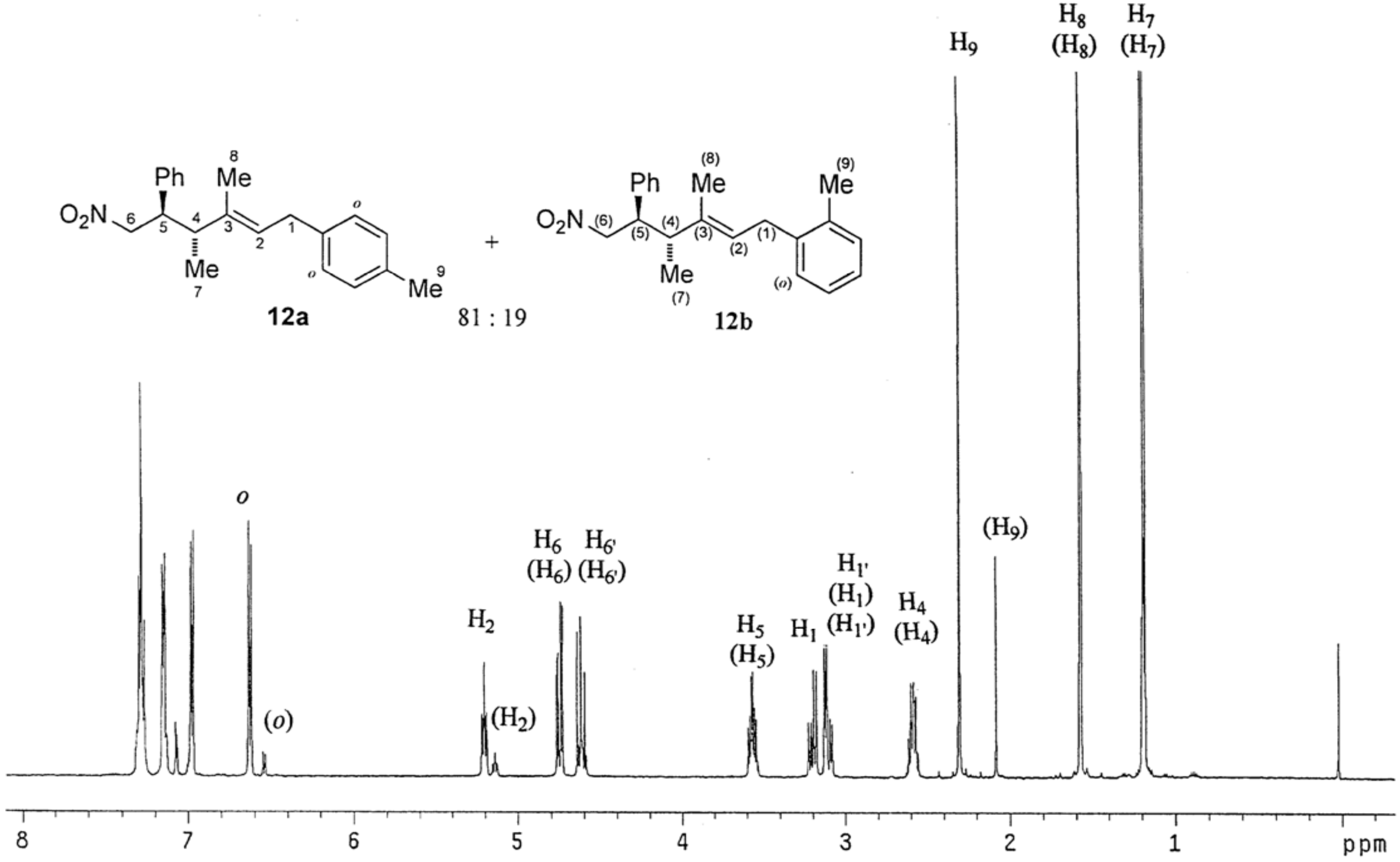

^{1}H NMR Spectrum

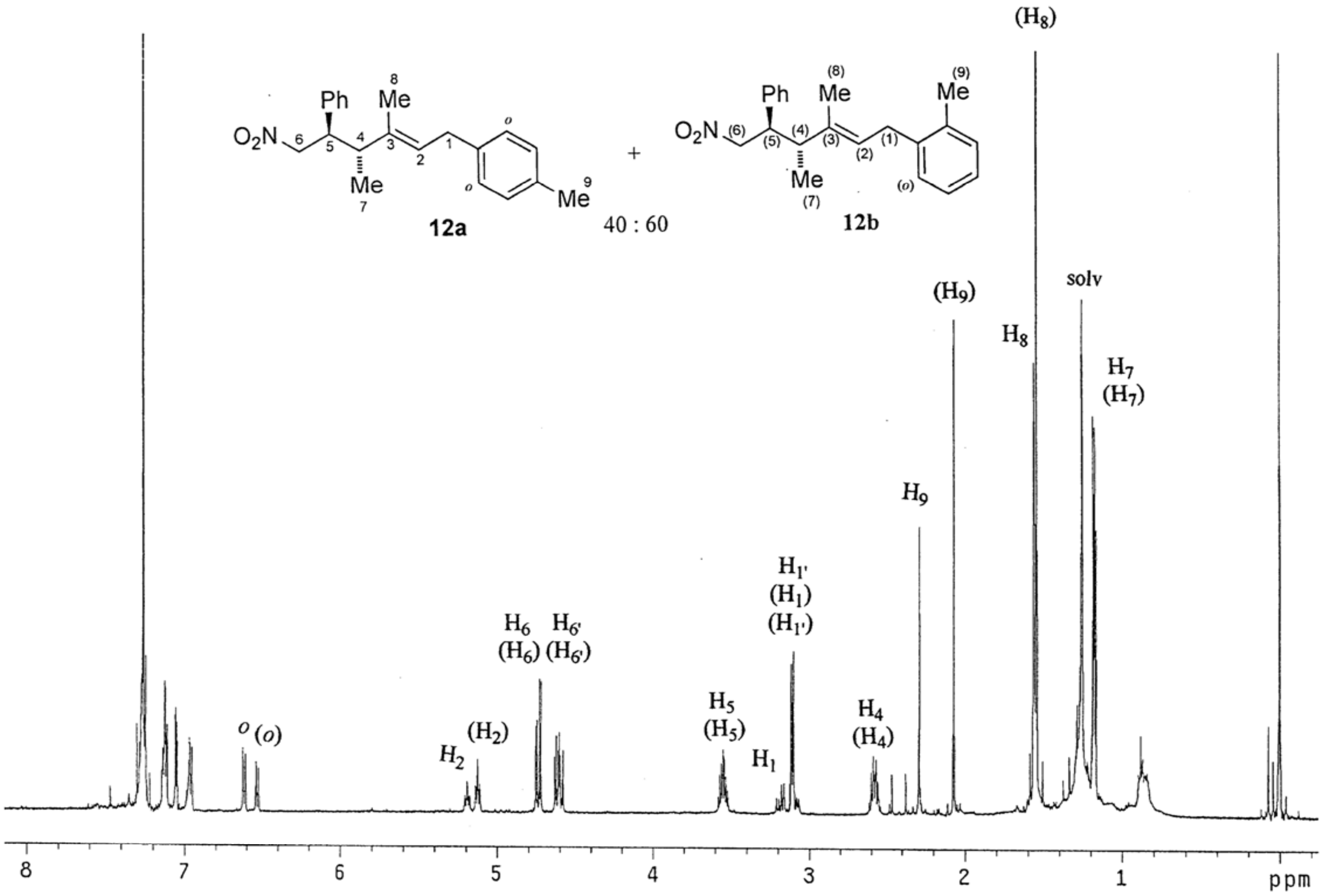

^{1}H NMR Spectrum

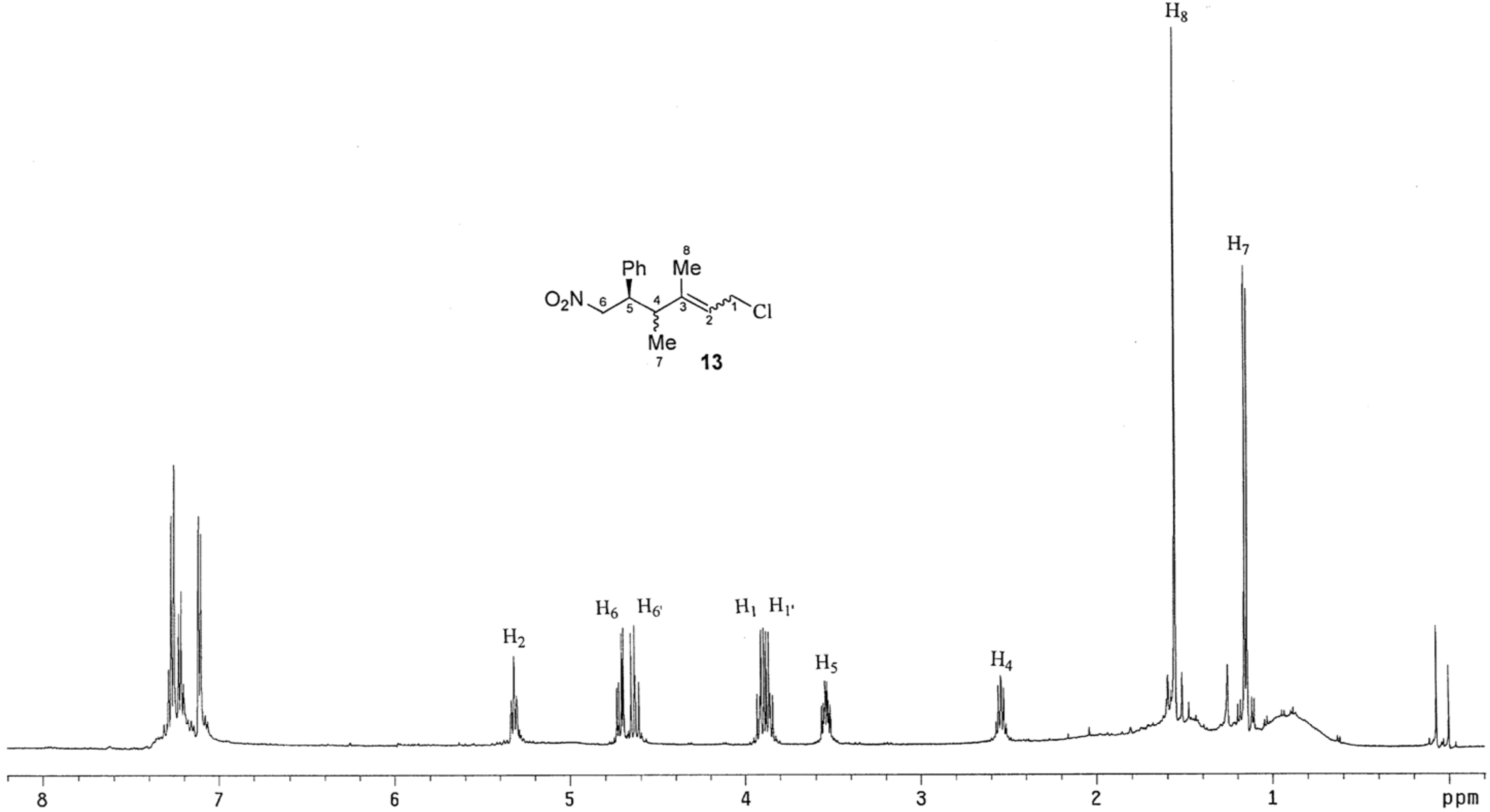

^{1}H NMR Spectrum

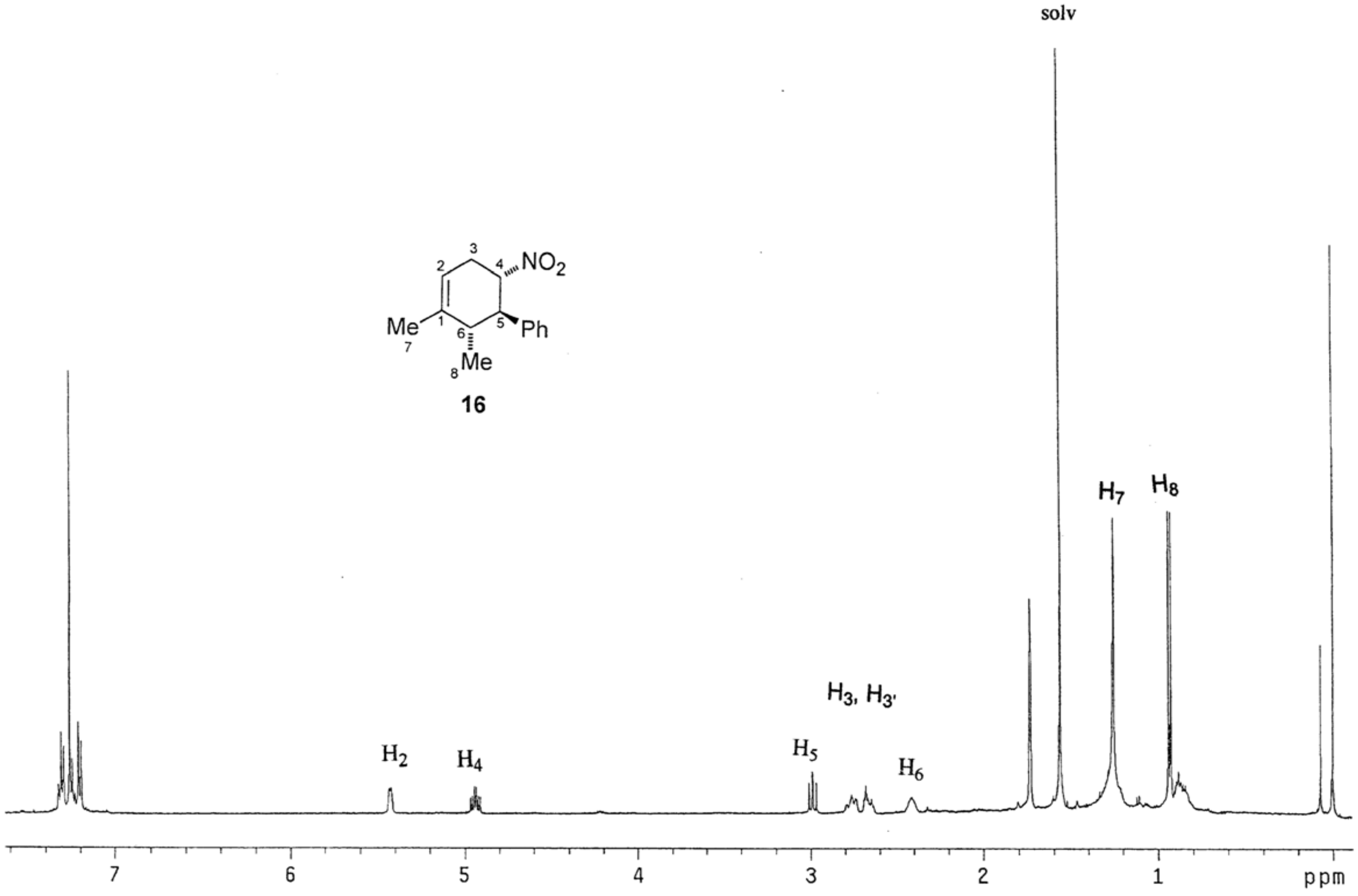

^1H NMR Spectrum

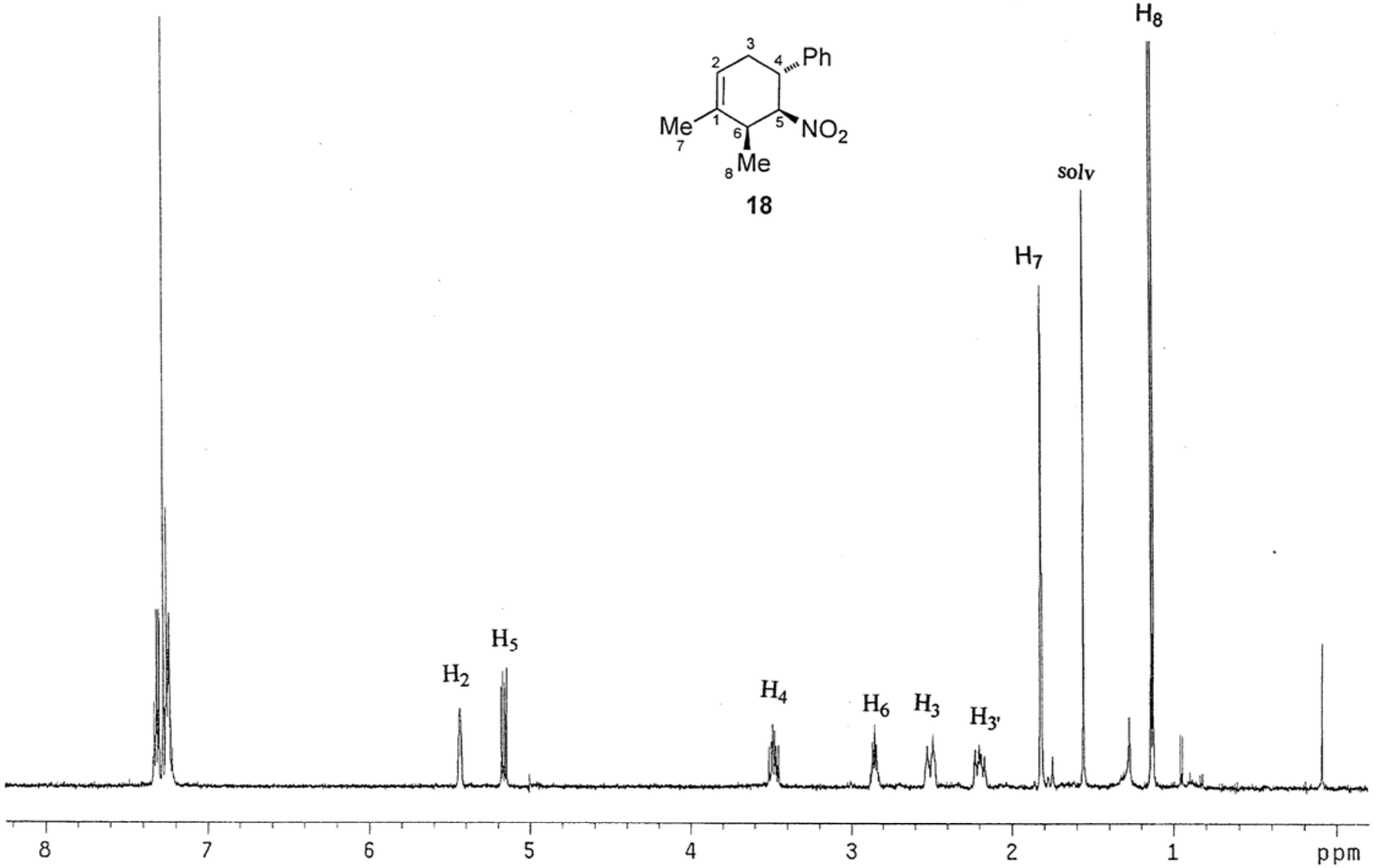

^{1}H NMR Spectrum

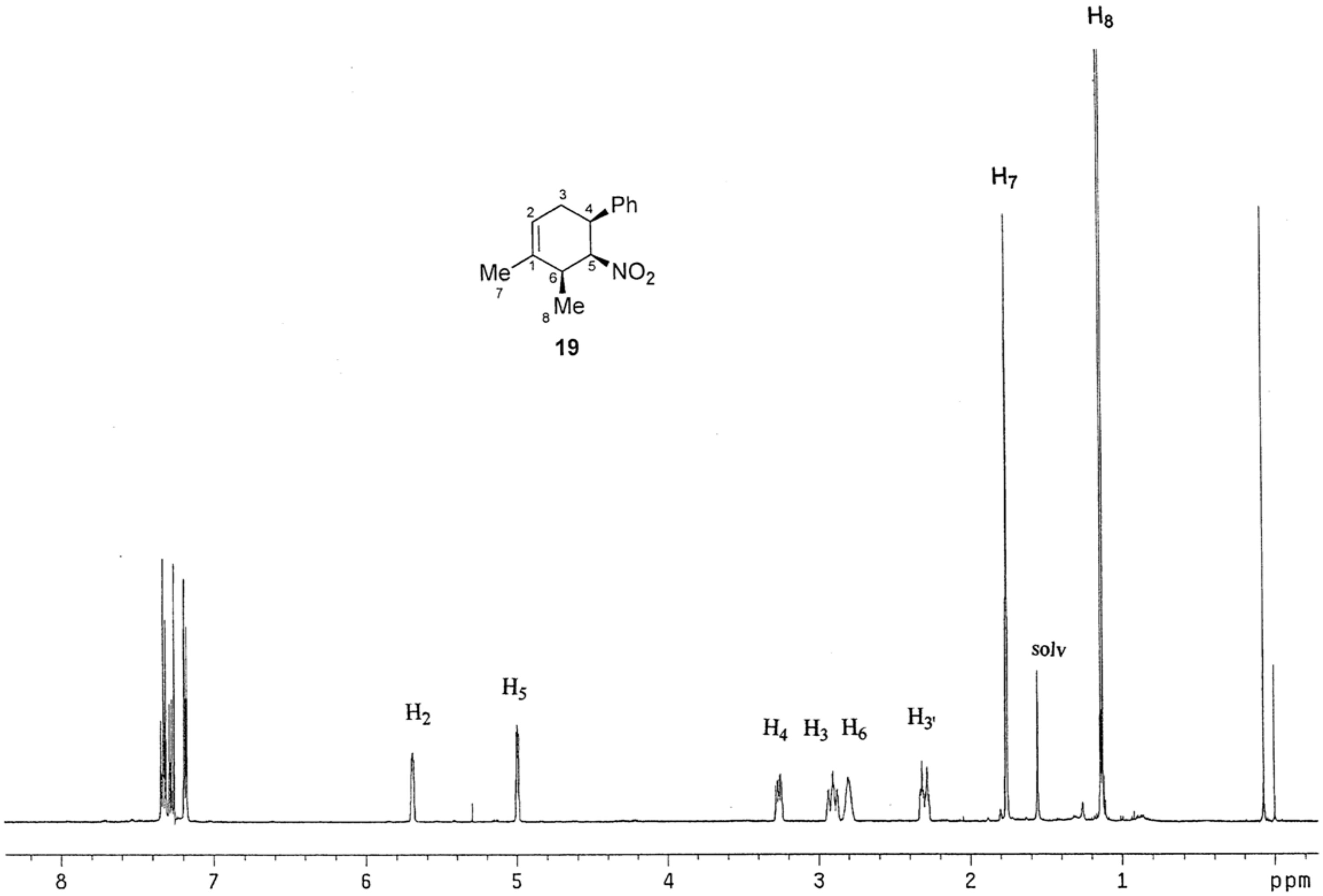

^{1}H NMR Spectrum

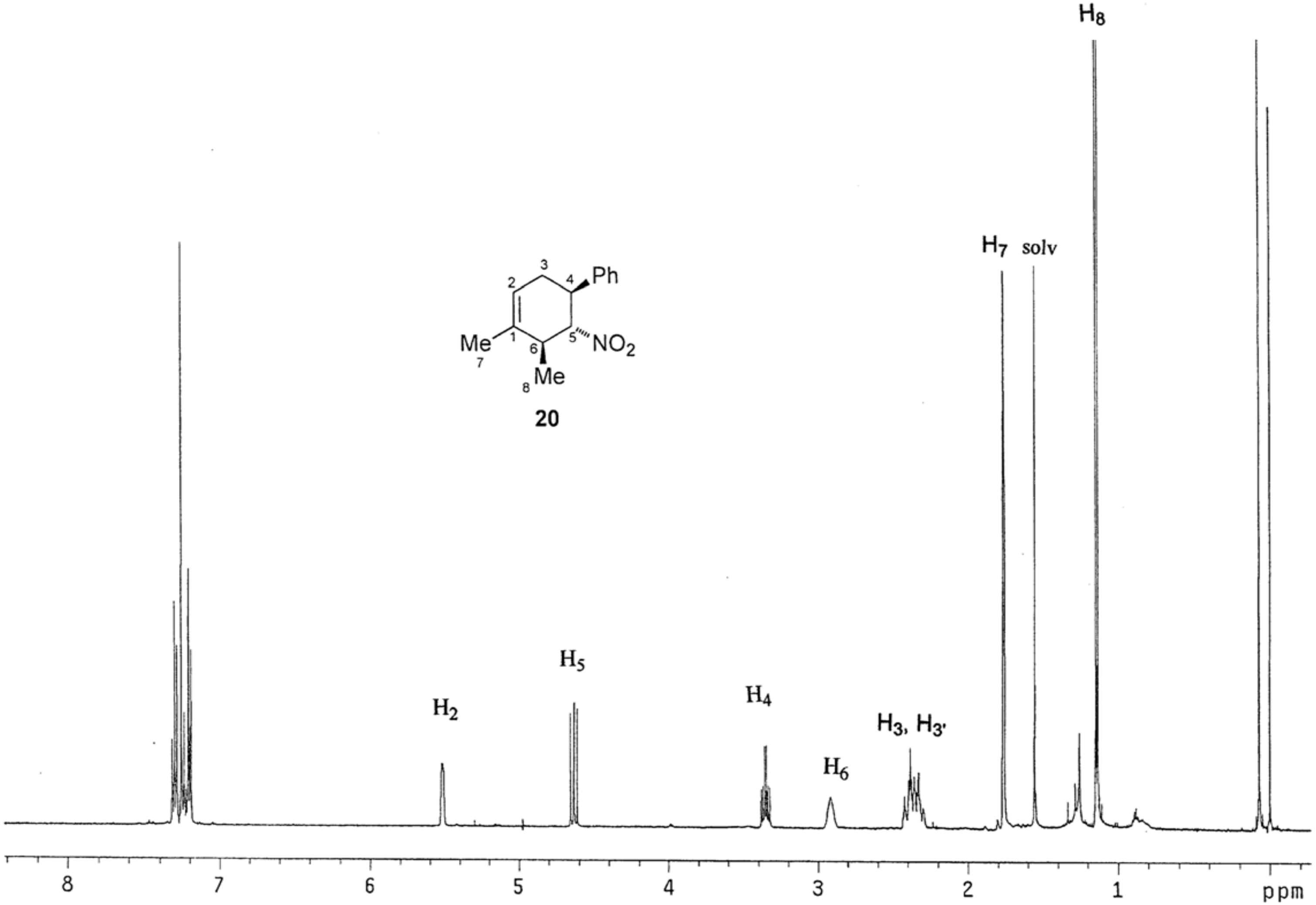

^{13}C NMR Spectrum

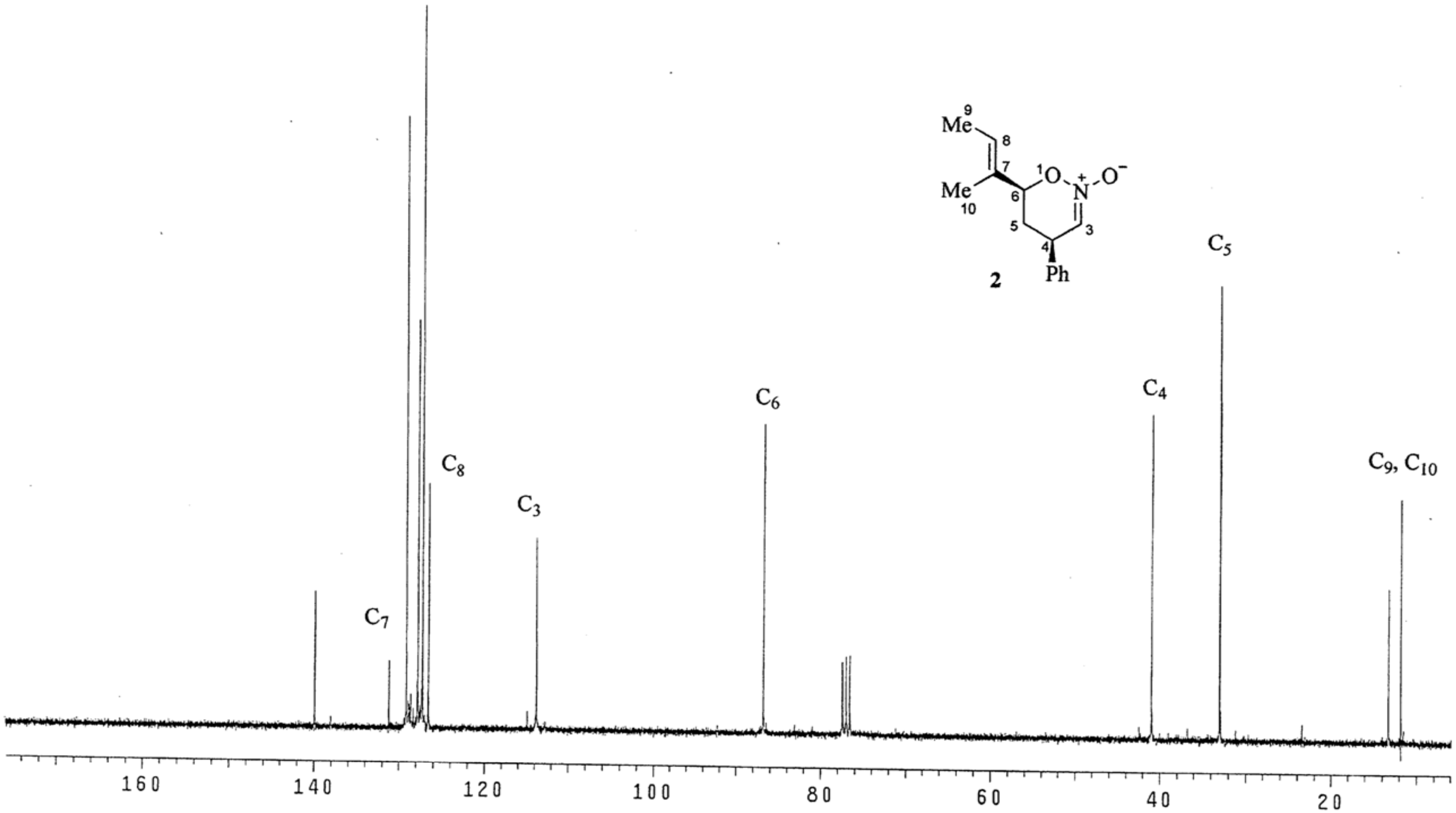

^{13}C NMR Spectrum

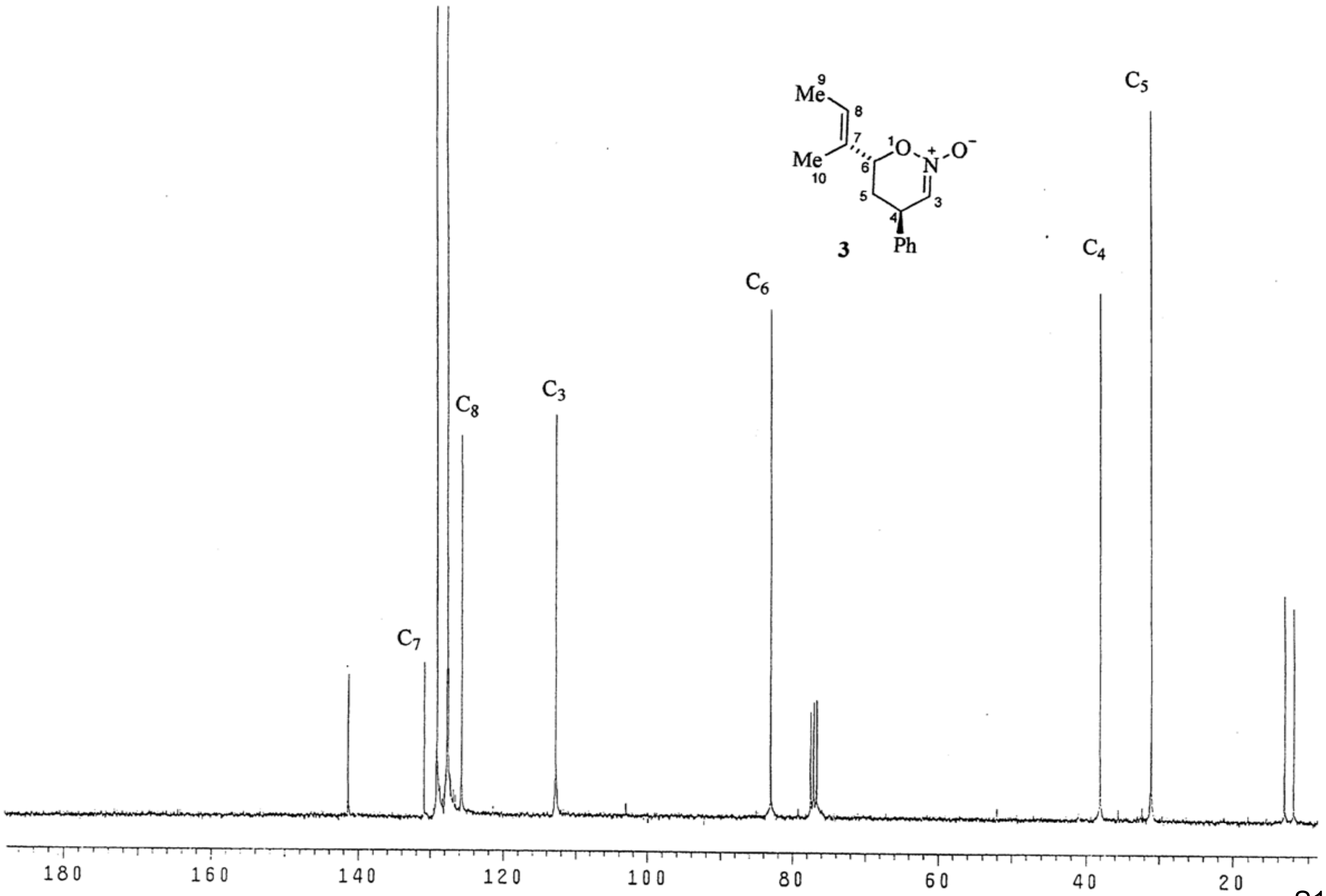

^{13}C NMR Spectrum

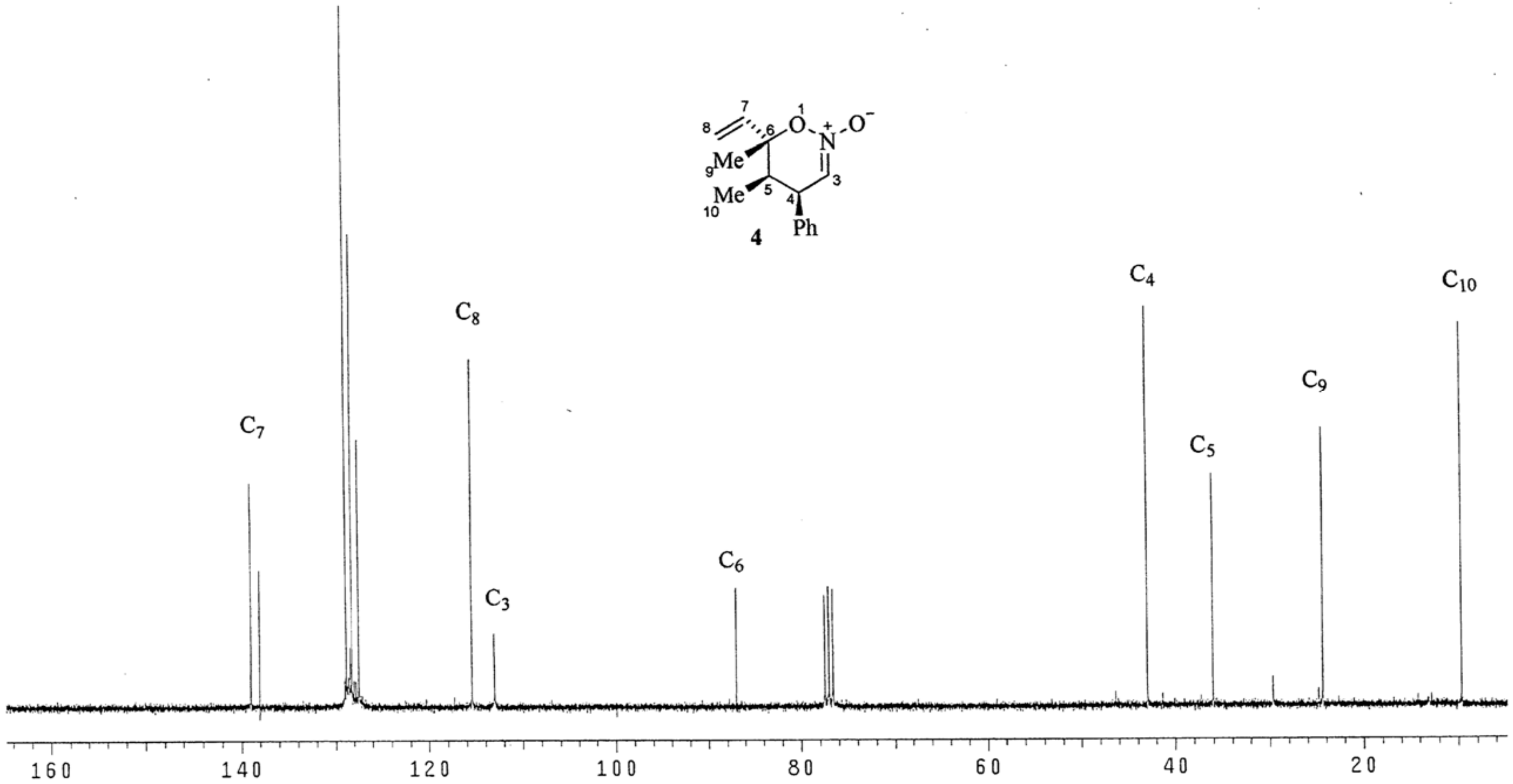

^{13}C NMR Spectrum

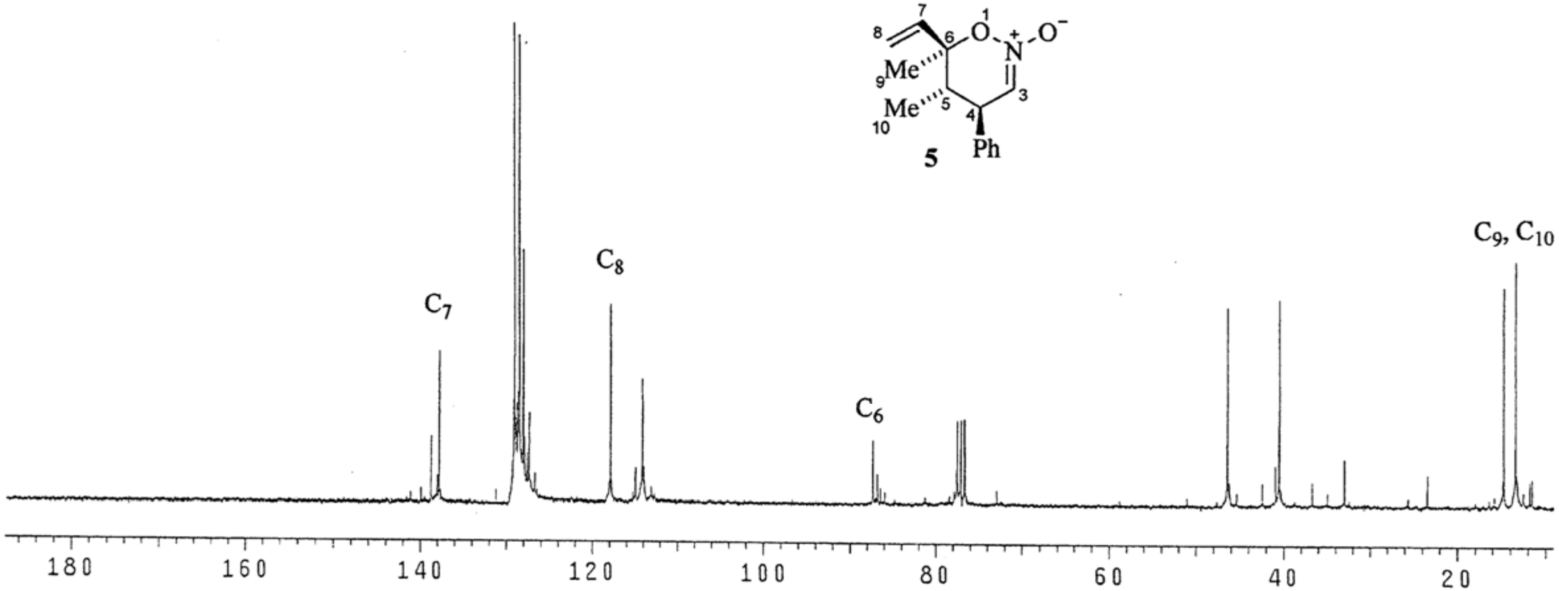

^{13}C NMR Spectrum

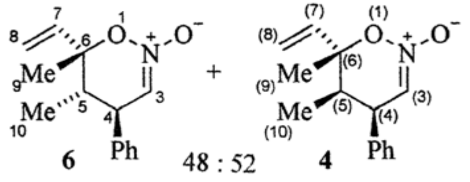

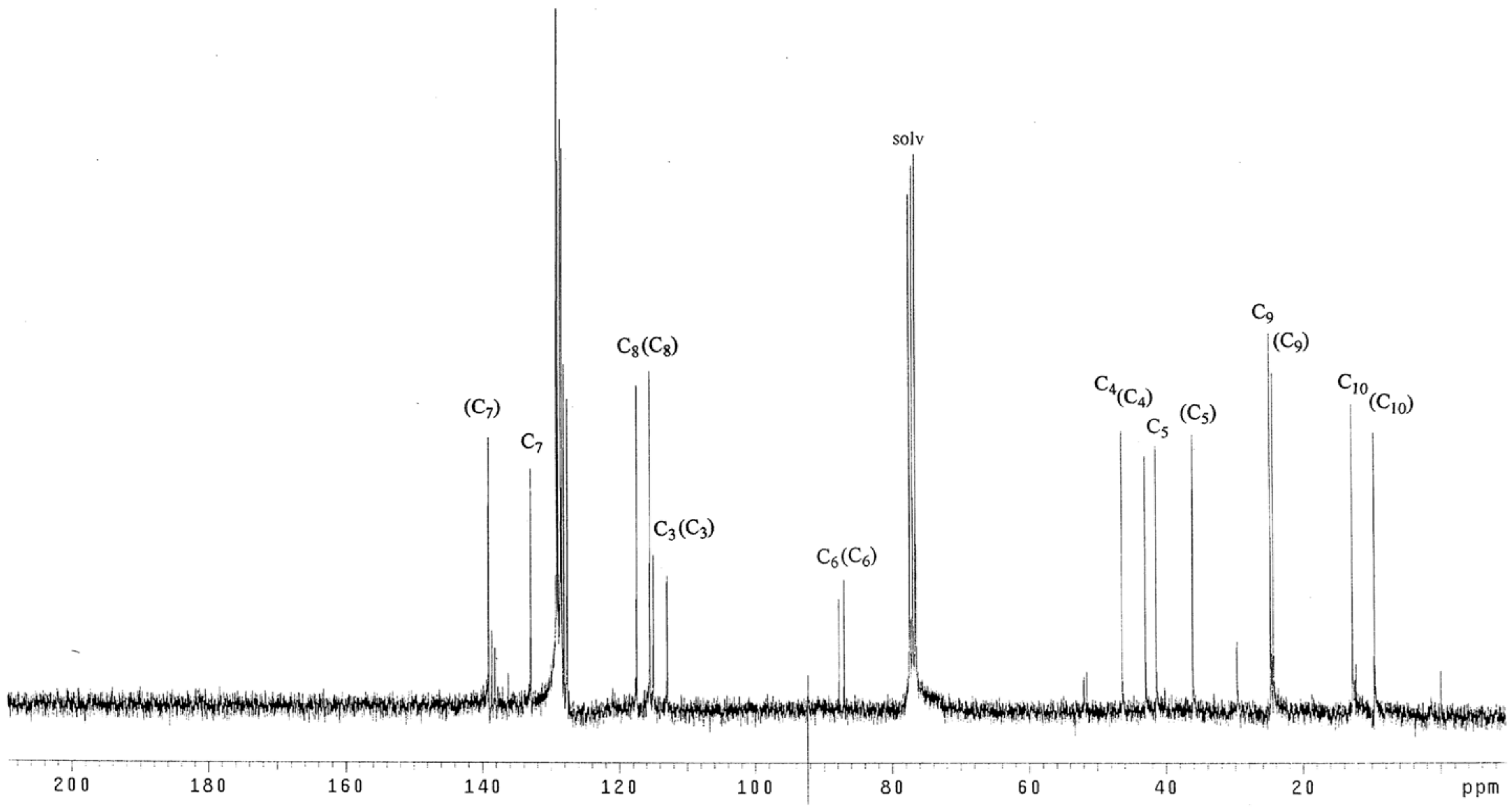

^{13}C NMR Spectrum

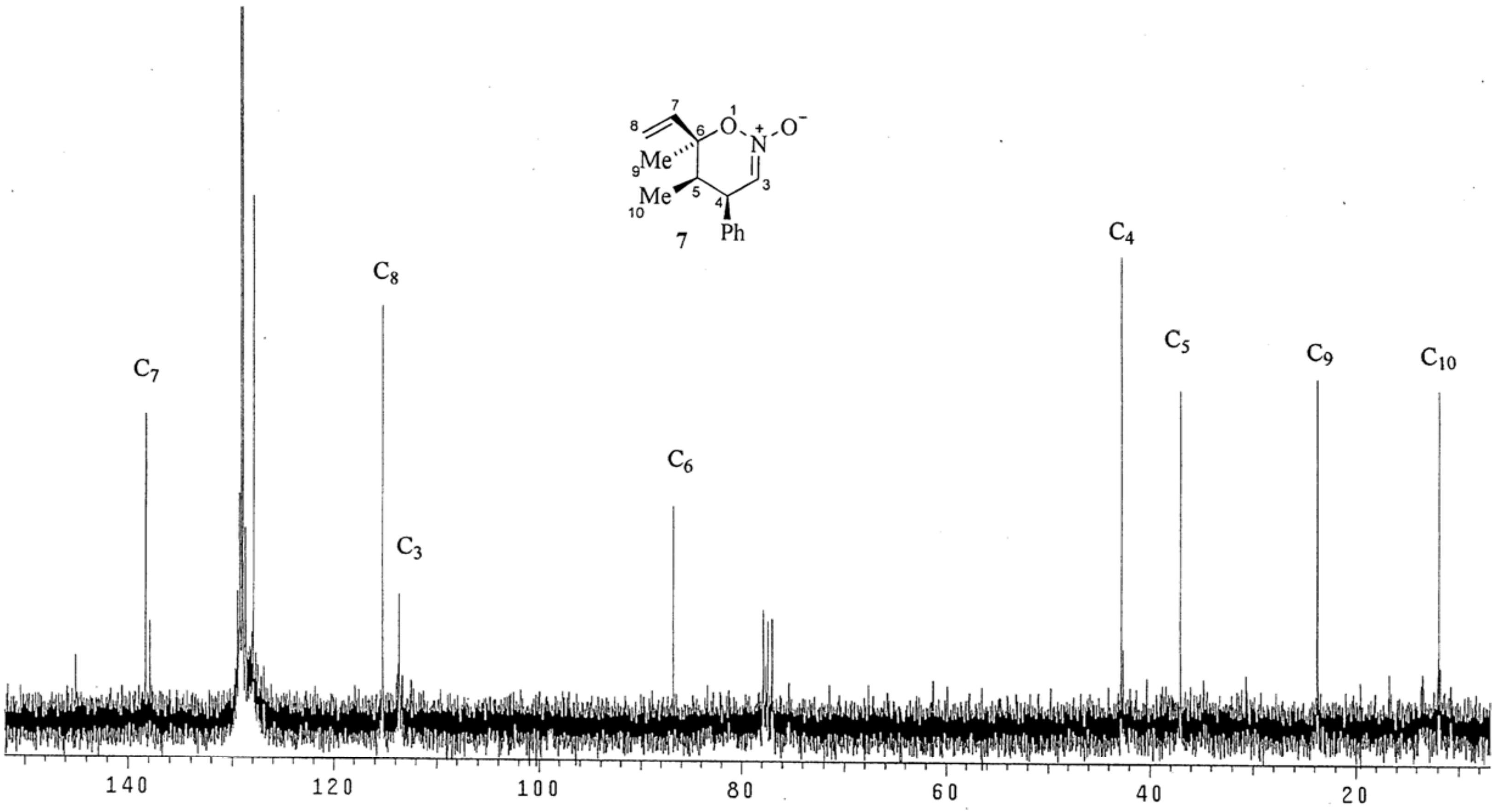

¹³C NMR Spectrum

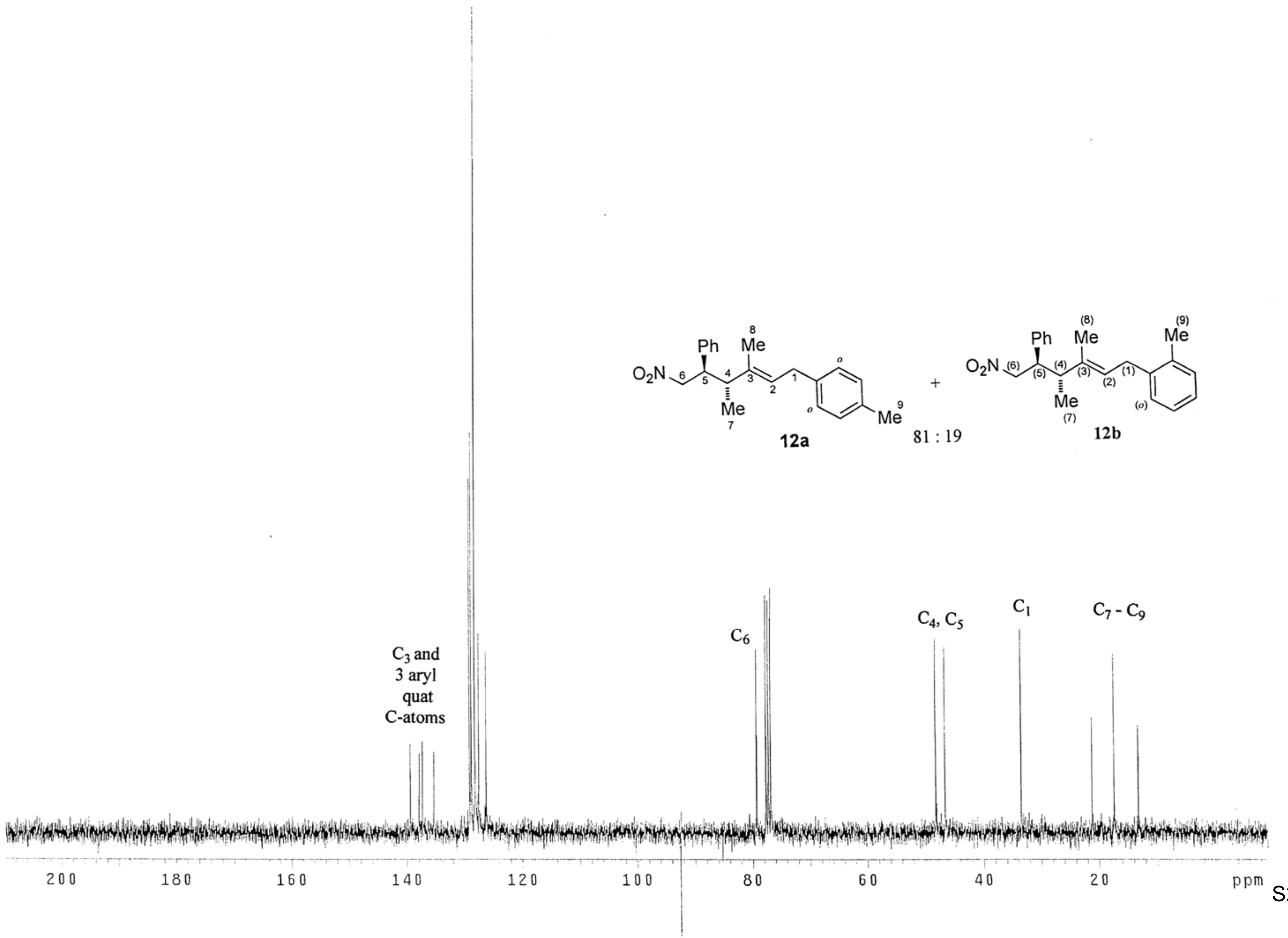

^{13}C NMR Spectrum

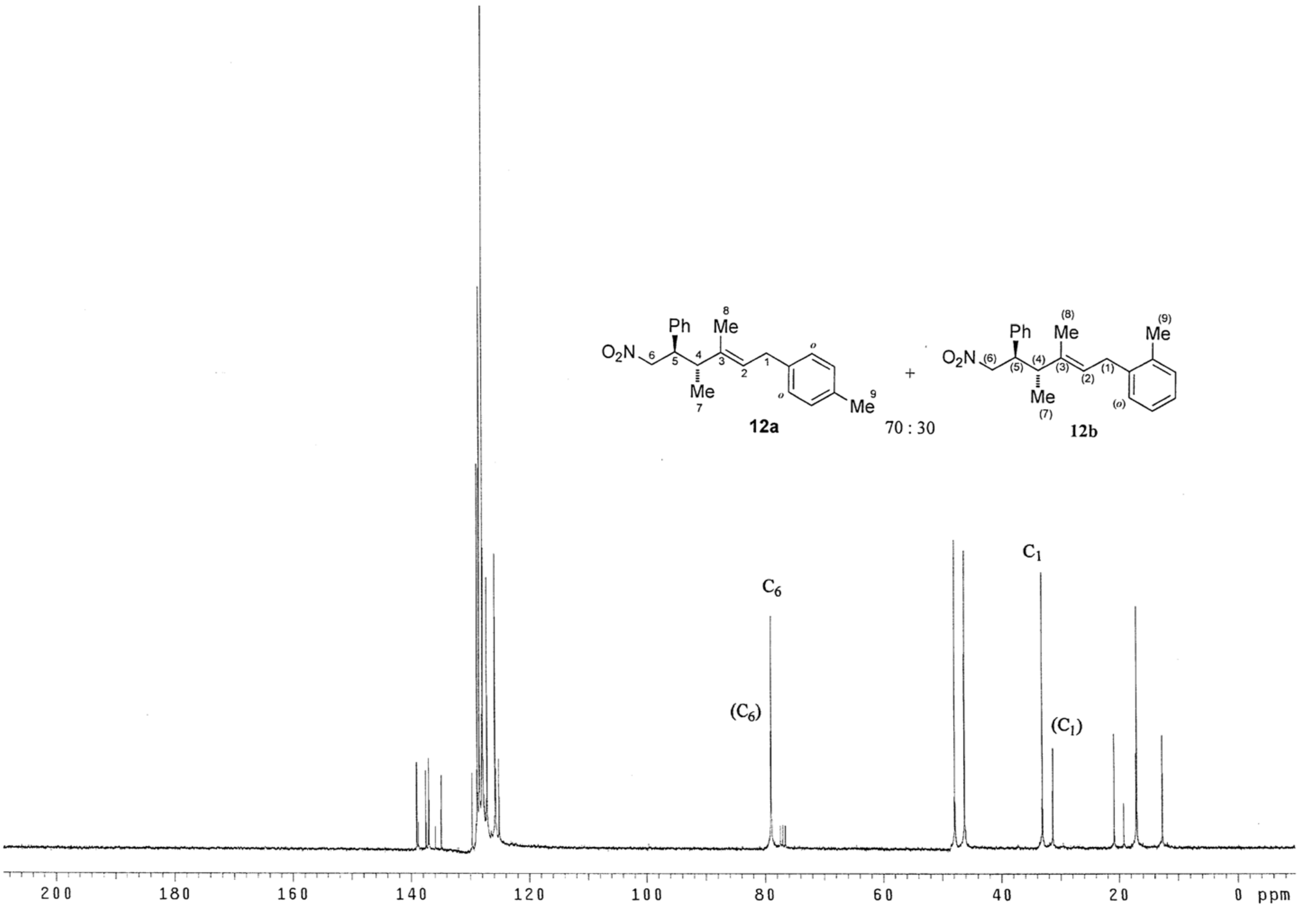

^{13}C NMR Spectrum

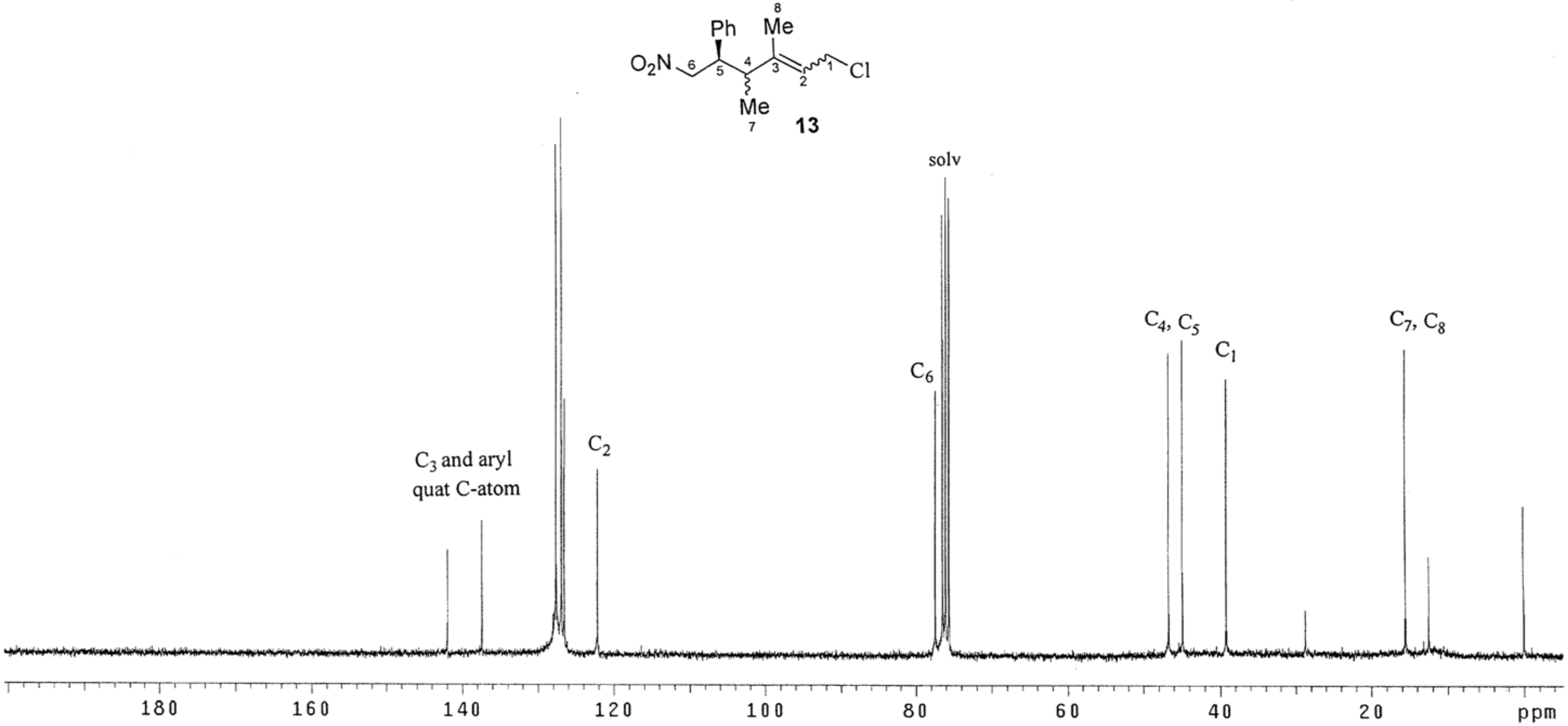

^{13}C NMR Spectrum

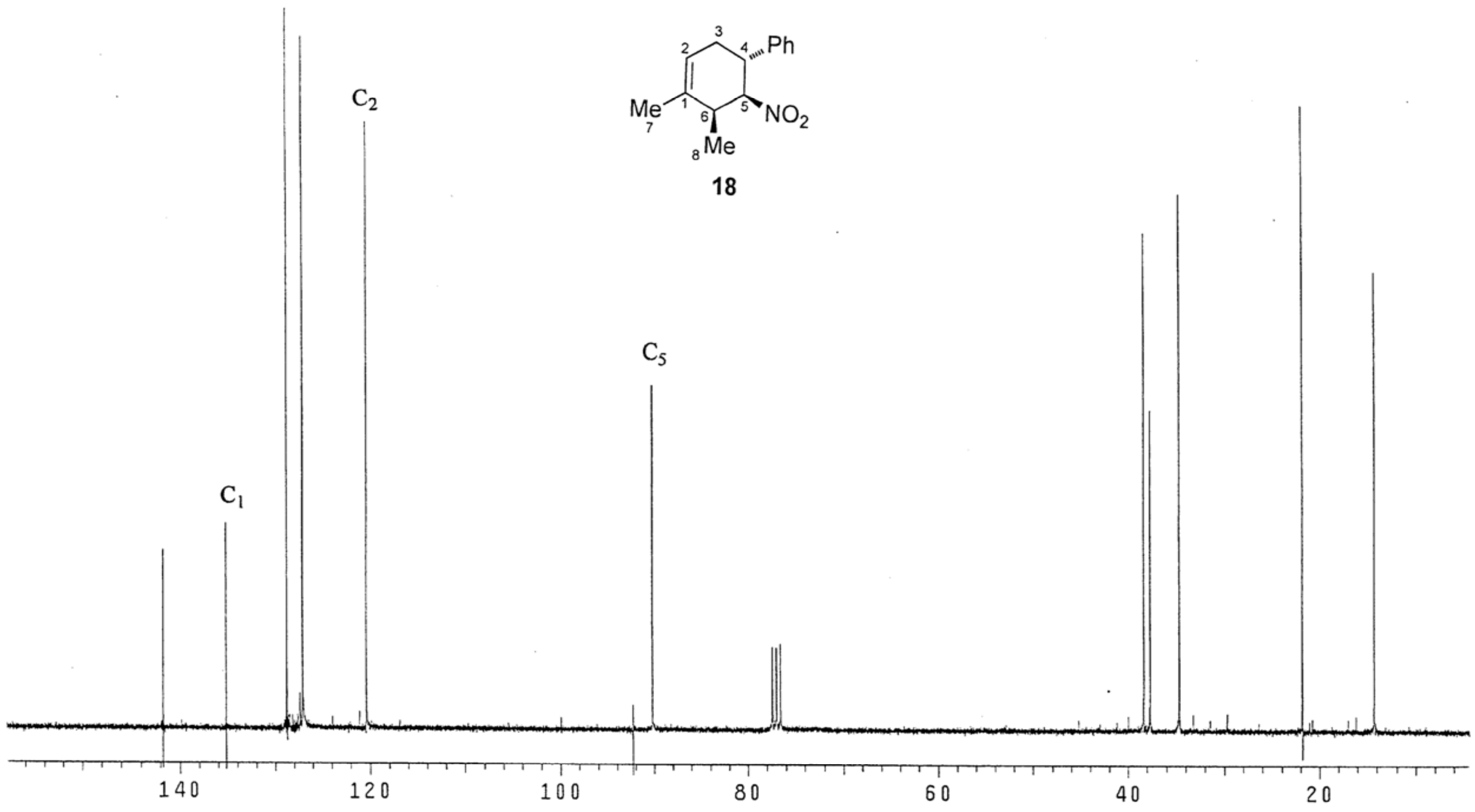

^{13}C NMR Spectrum

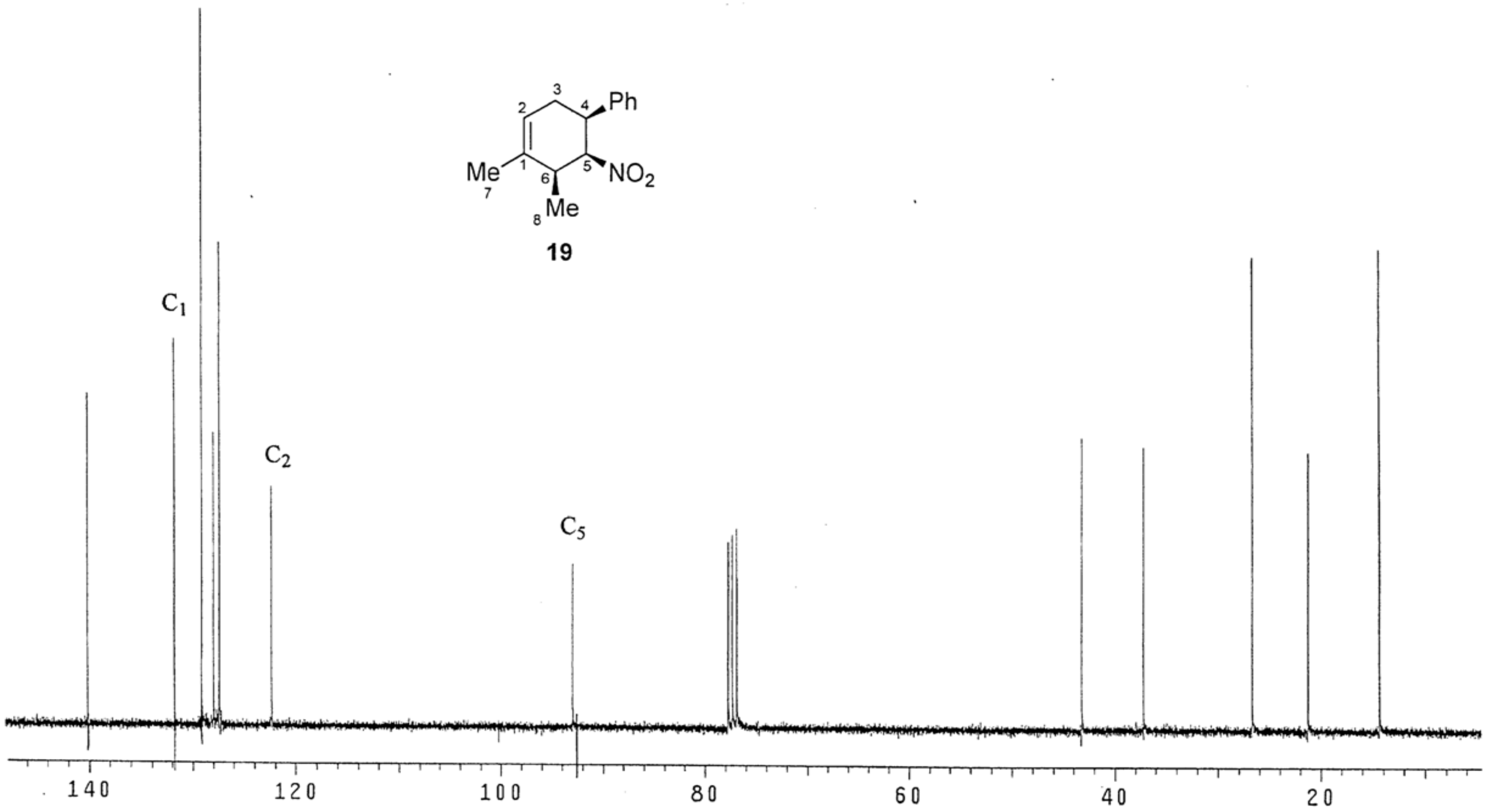

^{13}C NMR Spectrum

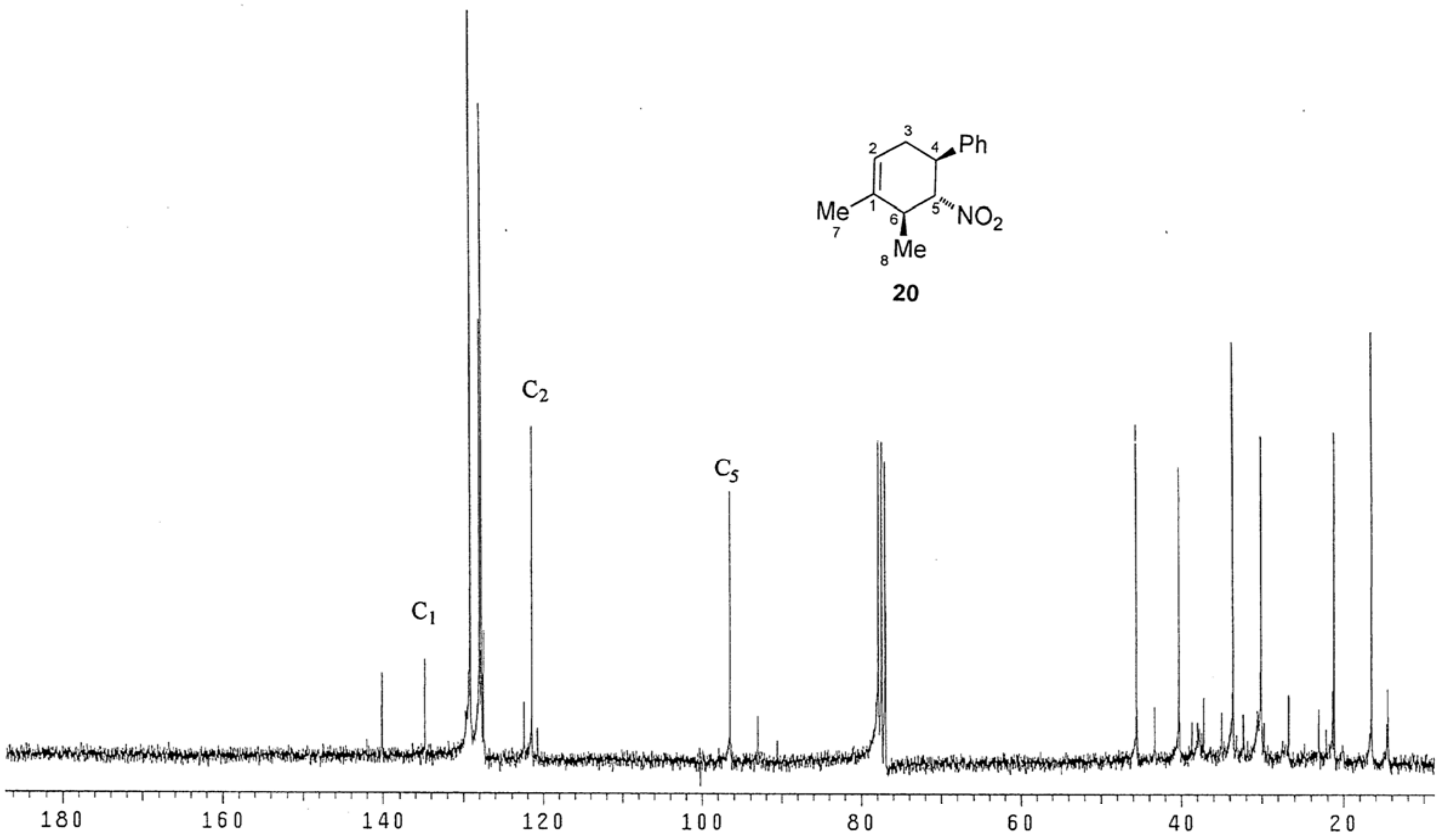

DEPT Spectrum

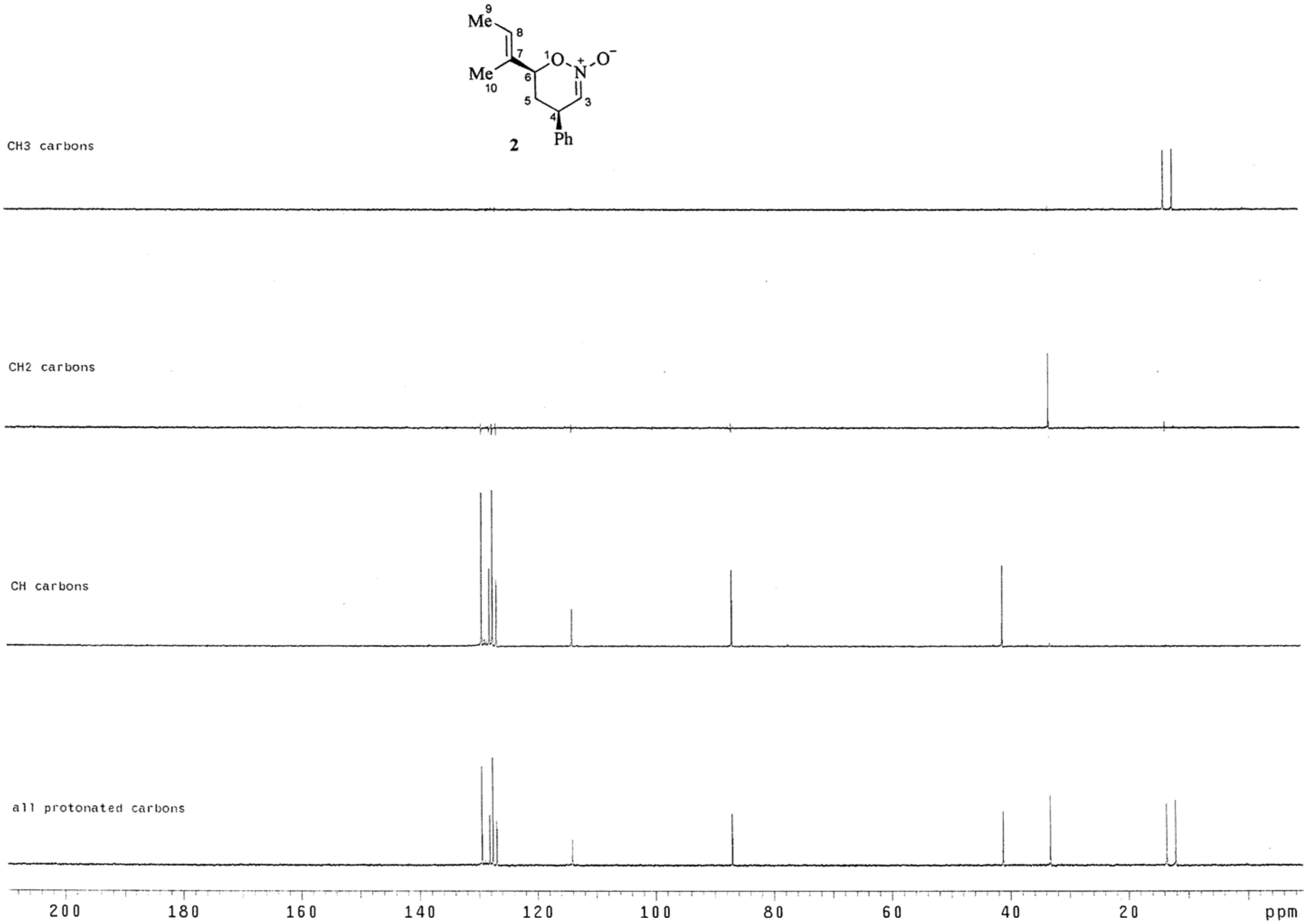

DEPT Spectrum

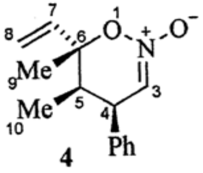

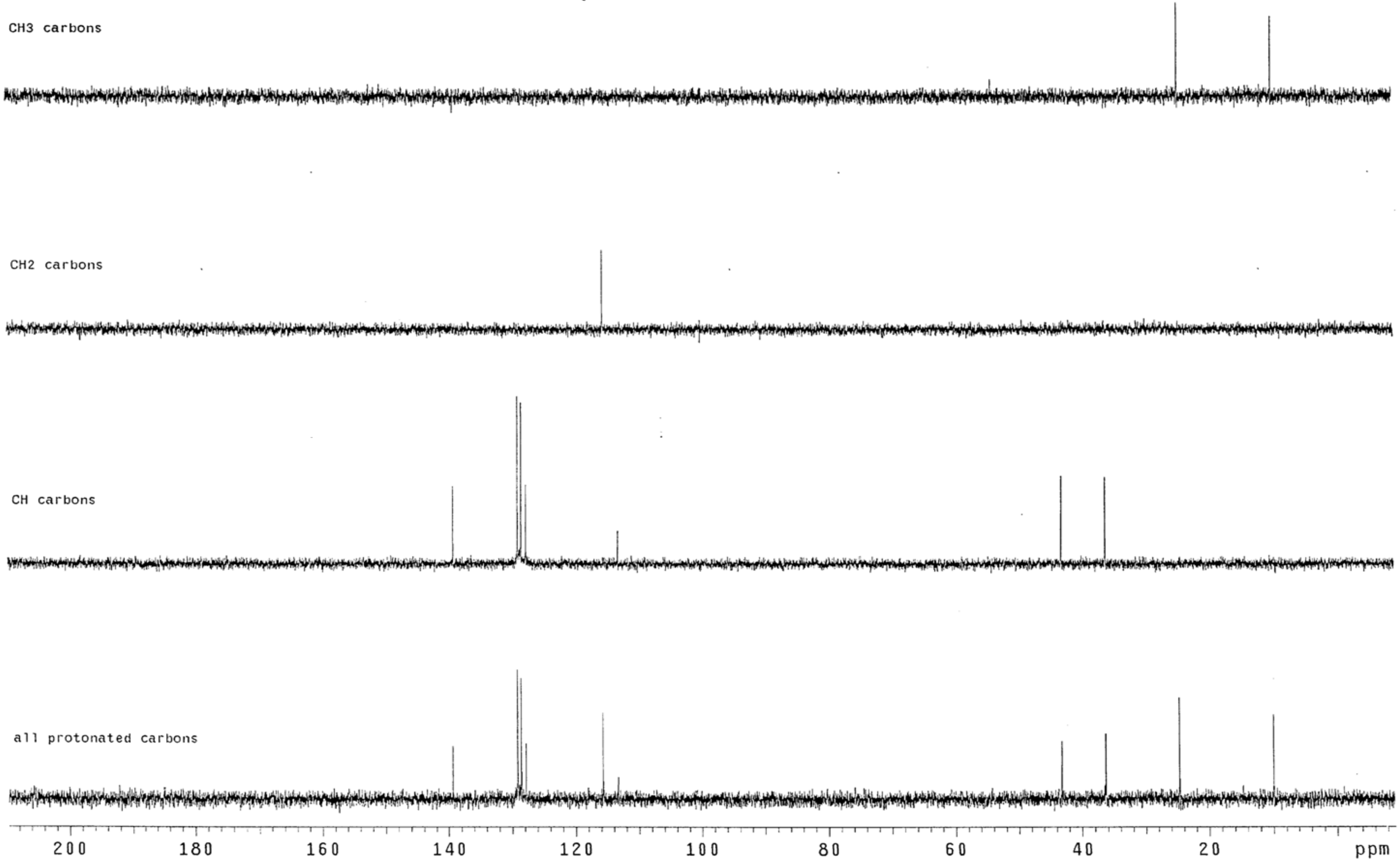

DEPT Spectrum

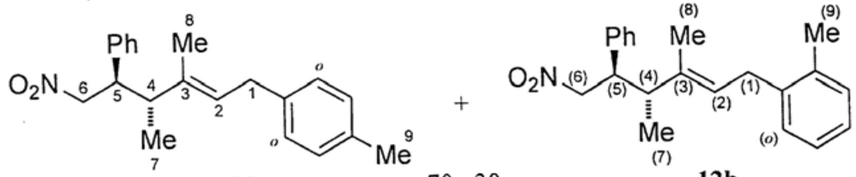

12a 70 : 30 **12b**

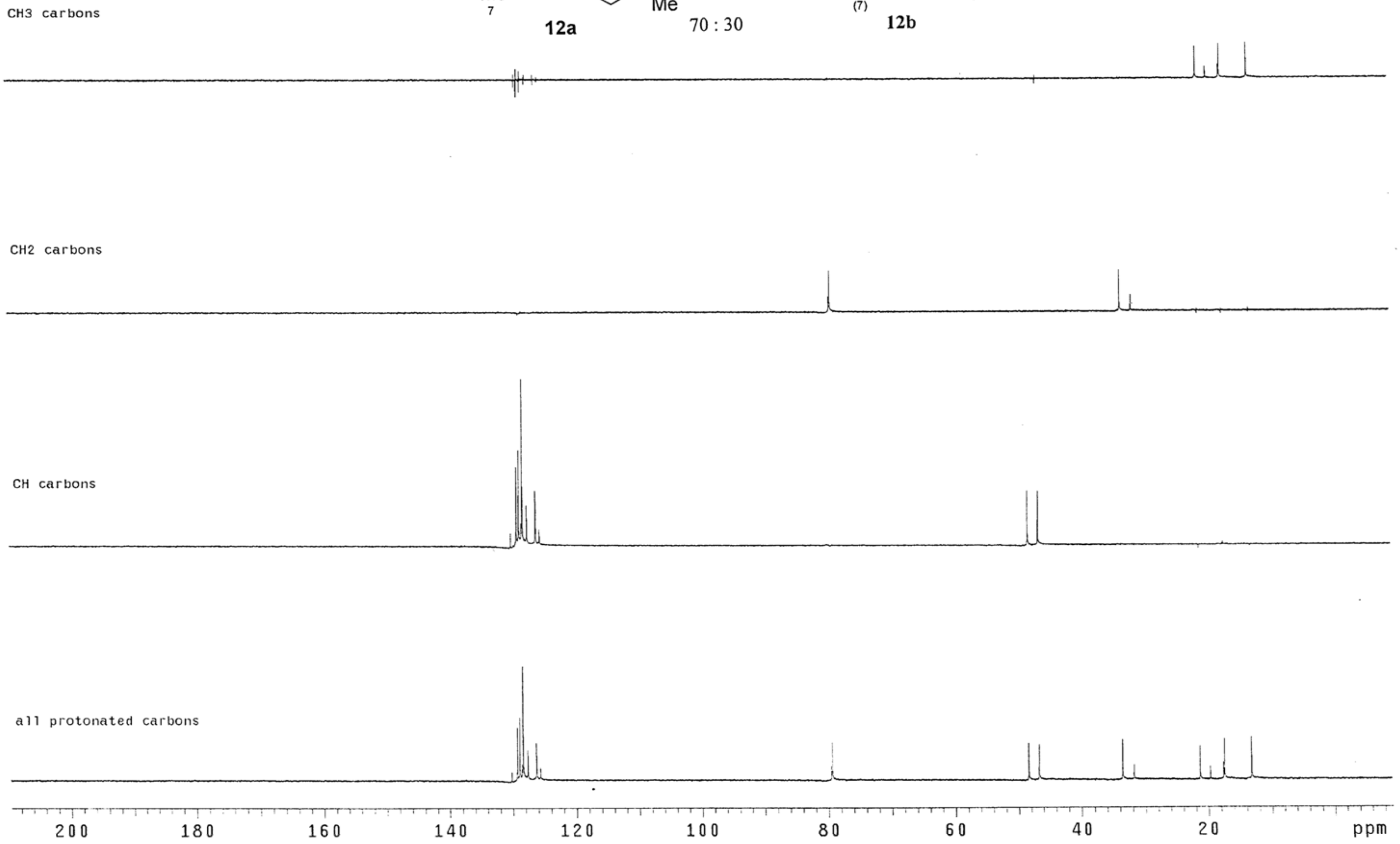

Supplement: File 2 — NMR spectra and signal assignments for compounds 2–7, 12,13, and 18–20. [file Beilstein_J_Org_Chem-09-2137-s002.pdf]
